# Use of PGPB in bio-fertilisation: preserving the soil microbiome and enhancing field production of alfalfa

**DOI:** 10.3389/fmicb.2025.1735729

**Published:** 2026-01-20

**Authors:** Vanesa Mercedes Fernández-Pastrana, Marina Robas Mora, Daniel González-Reguero, Agustín Probanza, Diana Penalba Iglesias, Pedro Antonio Jiménez Gómez

**Affiliations:** Department of Pharmaceutical Science and Health, CEU San Pablo University, Montepríncipe Campus, Madrid, Spain

**Keywords:** alfalfa (*Medicago sativa*), biofertilisers, PGPB (*Bacillus pseudomonas*), soil antibiotic resistance, valorised organic residues

## Abstract

Sustainable fodder production requires fertilisers that increase yield without compromising soil ecology. We tested whether a humic-rich biofertiliser derived from valorised horticultural waste (ORGAON® PK) could be enhanced with two genomically screened plant-growth-promoting bacteria (PGPB) in a field trial with *Medicago sativa*. The crude residue substantially increased biomass, and the addition of Bacillus sp. C1 or Pseudomonas sp. C2 further redirected these gains towards improved fibre digestibility or greater protein and energy content. Soil microbial diversity was maintained, although community composition shifted towards taxa involved in organic-matter degradation and nitrification. Both inoculants persisted without displacing dominant native genera, and biofertilised soils showed reduced susceptibility to *β*-lactam antibiotics. Overall, pairing OPK with targeted PGPB enhanced forage yield and quality while supporting microbiome resilience, highlighting a promising One-Health-aligned alternative to mineral fertilisers. Multi-season trials are now needed to validate broader applicability.

## Graphical abstract

**Figure:**
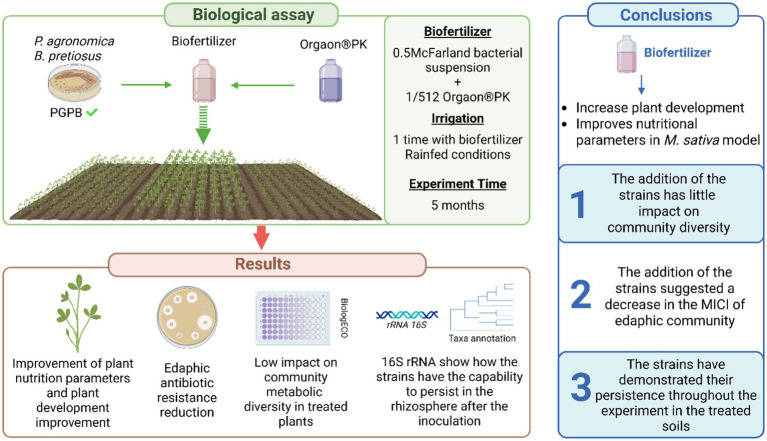

## Highlights

- Crude ORGAON® PK + *Bacillus* sp. C1 doubled alfalfa dry-matter yield (+101%) in field.
- *Bacillus* sp. C1 raised fibre digestibility (+6 pp); *Pseudomonas* sp. *C2 lifted protein (+3%).*
- Taxon turnover enriched Chitinophaga and Variovorax enhancing C-N cycling, but kept Shannon stable, showing resilience.
- Lower resistance phenotype with PGPB inoculants: *β*-lactam MICs fell 19–30 mg L^−1^ in all biofertilised soils.

## 1 Introduction

The United Nations Sustainable Development Goal “Zero Hunger” (Arora and Mishra, 2022) highlights the need to reconcile environmental stewardship with a secure supply of food for both humans and livestock. Yet soil degradation already impairs between 25 and 40% of the planet’s land surface, placing future food security at risk (Prăvălie et al., 2024; Schillaci et al., 2023).

Among forage crops, alfalfa (*Medicago sativa* L.) is particularly valuable. Its symbiosis with nitrogen-fixing rhizobia enriches soil fertility, while its high protein content underpins animal production (Qin et al., 2024; Fan et al., 2023; Xu et al., 2024). However, productivity is threatened by salinisation, drought, deforestation and erosion, as documented in the global assessment of soil degradation (FAO and UNEP, 2021; Goswami et al., 2016).

Mineral fertilisers are widely deployed to offset these losses, but their over-use carries environmental penalties; manufacture and application account for about 2.1% of global greenhouse-gas emissions (Menegat et al., 2022). At the same time, agriculture generates almost 974 Mt. yr.^−1^ of organic residues whose sustainable reuse could improve soil structure and deliver nutrients more efficiently than synthetic inputs (Atinkut et al., 2020; Feng et al., 2025). Indeed, nitrogen-use efficiency from organic amendments can approach 80%, compared with roughly 35% for mineral fertilisers (Yi et al., 2010; Rafique et al., 2025).

In Almería (Spain)—the world’s largest contiguous greenhouse area, covering 30,456 ha—vast quantities of horticultural waste accumulate annually (Pinna-Hernández et al., 2020). Valorising this biomass fits squarely within the circular-economy paradigm, returning organic matter to production systems without compromising food safety (Devi et al., 2025; Kanter et al., 2020; King, 2022). Because nutrients in raw waste are often locked in recalcitrant forms, their biotransformation by decomposer microorganisms has prompted the development of next-generation biofertilisers (Dasgan et al., 2022; Wu et al., 2022; Dzvene and Chiduza, 2024; Nabati et al., 2025).

Within this context, strains of *Bacillus* and *Pseudomonas* are prominent plant-growth-promoting rhizobacteria (PGPB): they solubilise nutrients, synthesise phytohormones and improve plant biometric and physiological traits (Giannelli et al., 2022; Hang et al., 2022; Vidal et al., 2022; Alattas et al., 2024; Huiqiong et al., 2024). They also enhance abiotic-stress tolerance, water retention and biocontrol of pathogens (Bakaeva et al., 2022; Pang et al., 2022; Schillaci et al., 2022; Buqori et al., 2025; Garrido-Sanz and Keel, 2025), although most evidence derives from controlled environments rather than field conditions.

High-throughput sequencing of *16S rRNA* amplicons now permits detailed monitoring of inoculant persistence and shifts in *α*- and *β*-diversity under realistic agronomic settings (Ma et al., 2023; Li et al., 2021). Nevertheless, PGPB deployment must be assessed through a One-Health lens, as some strains harbour mobile antibiotic-resistance genes (ARGs) (Pérez-Valera et al., 2019; Nguyen et al., 2023; Pagaling et al., 2023). Selecting inoculants that lack virulence factors and transferable ARGs is therefore essential (Djordjevic et al., 2024; Musicha et al., 2024).

This work investigates the potential of ORGAON® PK (OPK)—a biofertiliser derived from valorised horticultural waste—to enhance, simultaneously, the yield and nutritional quality of field-grown *Medicago sativa*. We compared the organic amendment alone, its sterilised counterpart and a mineral fertiliser, each applied with or without two benchmark PGPB: *B. pretiosus* (C1) and *P. agronomica* (C2). Specifically: (i) biomass and key forage attributes (fibre digestibility, crude protein, amino-acid profile and water-soluble carbohydrates); (ii) structural and functional responses of the rhizosphere microbiome (*α*/β-diversity, metabolic activity and taxonomic composition); and (iii) changes in soil antibiotic-resistance phenotypes was assessed.

We formulated a primary, testable hypothesis: that combining OPK with genomically vetted PGPB would increase alfalfa biomass yield and nutritional quality without reducing soil microbial diversity or disrupting rhizosphere ecological functioning. Secondary analyses (e.g., KEGG-orthologue predictions, fine-scale taxonomic turnover, and antibiotic-resistance phenotypes) were included to contextualise the main response under field conditions.

The overarching goal is to provide robust field evidence positioning residue-based biofertilisers fortified with PGPB as high-performance, low-risk alternatives to conventional mineral fertilisation.

## 2 Experimental procedures

### 2.1 Biological treatments: PGPB strains

Two plant-growth-promoting rhizobacteria were employed: C1 (*Bacillus* sp.; proposed as “*Bacillus pretiosus* sp. nov.” in Robas-Mora et al., 2023; not validly published under the ICNP; type strain DSM 114702^T^, CECT 30674^T^) and C2 (*Pseudomonas* sp.; proposed as “*Pseudomonas agronomica* sp. nov.” in Robas-Mora et al., 2023; not validly published under the ICNP; type strain DSM 114959^T, CECT 30673^T). Both strains were previously isolated from the rhizosphere of a wild *Medicago sativa* (L.) plant collected in the Almadén Mining District (Ciudad Real, Spain) and selected according to their Biomercurorremediator Suitability Index (IIBMR) following Robas-Mora et al. (2023). This index is a composite score (0 to 1) that integrates (i) intrinsic Hg^2+^ tolerance (minimum inhibitory concentration), (ii) Hg bio-accumulation capacity and (iii) expression of plant-growth-promoting traits such as indole-3-acetic-acid production, ACC-deaminase activity and phosphate solubilisation. Strains with IIBMR ≥ 6 are considered suitable bioinoculants for crops grown on trace-metal-impacted soils (Robas-Mora et al., 2023). Full phenotypic, chemotaxonomic and genetic characterisation is provided in Supplementary Table S1.

### 2.2 Irrigation matrices

#### 2.2.1 Valorised organic fertiliser (OPK)

The organic fertiliser ORGAON® PK (OPK), previously described by Robas-Mora et al. (2023), was prepared from the leachate of horticultural residues supplemented with phosphorus (0.10 ± 0.03% w/w) and potassium (2.58 ± 1.52% w/w). The physicochemical properties of OPK are summarised in Supplementary Table S1. The product was applied at a 1: 512 dilution (OPK: H₂O).

To assess the effect of the organic matrix in the absence of its native microbiota, a sterilised variant (OPK_ST) was produced. Fifty millilitres of OPK were exposed to UV-C radiation (265 nm, Philips TUV 30 W G30T8) for 15 min in a laminar-flow cabinet; the fertiliser was distributed in Petri dishes to a depth < 5 mm to ensure uniform irradiation. Pre- and post-irradiation aliquots were analysed for humic extract, fulvic acids, DOC and inorganic N/P (Supplementary Table S2). No significant differences were detected (*t*-test, *p* > 0.05), confirming that the 15-min UV-C protocol removed viable microbiota while leaving the chemical profile essentially unchanged (Supplementary Table S3). Sterility was verified by plating 100 μL of the treated solution, in duplicate, on LB agar (Condalab®, Madrid, Spain) and incubating at 28 ± 1 °C for 72 h. Only samples showing no microbial growth in either replicate were deemed sterile. Sterilised solutions were stored at 4 °C and used within 24 h of preparation.

Prior to field application the residue-microbiome safety was screened. The crude residue (OPK) was assayed for potential human or environmental pathogens. Triplicate 10-mL aliquots were pelleted (10,000 × g, 10 min), the pellets were processed with the DNeasy PowerSoil Pro kit (QIAGEN) and the V3–V4 *16S-rRNA* region was sequenced as described above. After DADA2 denoising, amplicon sequence variants were classified against SILVA 138.99; additionally, all quality-filtered reads were mapped with Kraken2 (v2.1.3, max-hits = 1) against the NCBI RefSeq “bacteria, archaea & viruses” database to capture low-abundance taxa. Taxa were flagged as putative pathogens if they belonged to any of the following genera: *Escherichia/Shigella, Salmonella, Klebsiella, Enterobacter, Citrobacter, Listeria, Staphylococcus, Pseudomonas aeruginosa* group, *Clostridium* sensu stricto 1, *Vibrio* or *Mycobacterium tuberculosis* complex. None of these genera exceeded 0.01% of total reads in any replicate (Supplementary Table S1).

The OPK microbiome was dominated by soil saprotrophs—Rubrobacter (18%), Nocardioides (9%), Streptomyces (7%) and Sphingomonas (4%)—plus Planctomycetota lineages typical of lignocellulose-rich residues (e.g., Pirellula, 3%). Absolute read counts for the 25 most abundant genera are shown in Supplementary Figure S1; together they accounted for 72% of the library, indicating moderate evenness. The combined Kraken2 screen and genus-level inspection confirmed the absence of clinically relevant pathogens at detectable levels, supporting the biosafety of OPK for open-field use.

#### 2.2.2 Chemical fertiliser (CF)

The inorganic reference fertiliser was a commercial NPK solution (Universal Fertilizer Complet®, COMPO Iberia S. L., Barcelona, Spain). Its guaranteed analysis is 7–5-6 (N-P₂O₅-K₂O) with micronutrients: 7% total nitrogen (2.1% nitrate-N, 1.3% ammoniacal-N, 3.6% urea-N), 6% water-soluble phosphorus (P₂O₅) and 5% water-soluble potassium (K₂O). The solution also supplies (all water-soluble): 0.01% boron (B), 0.002% copper (Cu), 0.02% iron (Fe), 0.01% manganese (Mn) and 0.002% zinc (Zn), each chelated with EDTA, together with 0.001% molybdenum (Mo).

#### 2.2.3 Biofertiliser formulation

The biofertiliser was prepared by enriching OPK with the selected PGPB strains (*B. pretiosus*—C1 or *P. agronomica*—C2) at the dilution recommended for fertigation of *M. sativa* (Robas-Mora et al., 2023). Twenty millilitres of a bacterial suspension adjusted to McFarland 4 were added per liter of OPK, yielding ≈ 10^8^ cfu mL^−1^ at 10^−1^ dilution. Fresh biofertiliser was prepared weekly and stored at 4 °C until use. The same inoculation protocol was applied to CF and to the water control (W).

#### 2.2.4 Irrigation regime

Each fertiliser was applied once, as the initial watering at the start of the trial. Thereafter, plants were maintained under rain-fed conditions from September 2022 to May 2023; no supplementary irrigation was provided. Two inoculations were performed, one at the beginning of the experiment in September and the second one before spring in February.

### 2.3 Experimental site and plant material

The field experiment was conducted in Villanueva de San Mancio, Valladolid, Spain (UTM zone 30 T, 333161 m E; 4,643,746 m N). Prior to treatment application, five composite cores (0–20 cm) were collected in a zig-zag pattern from each plot and pooled to obtain a representative sample of the site. Subsamples were air-dried, sieved (2 mm) and analysed at the CyTEMA (UCLM, Castilla la Mancha) following ISO-standard protocols: pH (1:2.5 H₂O; ISO 10390), electrical conductivity (ISO 11265), particle-size distribution (hydrometer), organic carbon (Walkley–Black), total nitrogen (Kjeldahl), CaCO₃ (Bernard calcimetry), Olsen P (ISO 11263) and exchangeable K (1 M NH₄OAc, AAS). The resulting clay-loam soil was slightly alkaline (pH 8.1), low in organic C (12.1 g kg^−1^) and moderately supplied with available P and K. Full physicochemical data are provided in Supplementary Table S4.

The crop was *Medicago sativa* L. cv. Aragón, established 3 years earlier under dry-land management (September–May). At inoculation (March) plants were ~10 cm tall and had previously received a single CF dressing. Harvest took place in May, 6 months post-inoculation.

Plots (25 m^2^) were arranged in a factorial design: four irrigation matrices (CF, OPK, OPK_ST, W) × three biological treatments (C0—no inoculant, C1—*B. pretiosus*, C2—*P. agronomica*). To preclude cross-contamination, plots were separated by 10 m buffer strips.

### 2.4 Extraction of rhizosphere microbial communities

Rhizosphere bacteria were recovered following García-Villaraco Velasco et al. (2009) with minor modifications. Briefly, 2 g of rhizosphere soil were suspended in 20 mL of sterile saline (0.45% w/v NaCl) and homogenised in an Omni-Mixer (Omni International, Kennesaw, USA) at 16000 rpm for 2 min to detach microorganisms from soil particles. The slurry was then centrifuged at 690 × g for 10 min in a refrigerated Mikro 22R centrifuge (Hettich, Gipuzkoa, Spain); the supernatant was retained for downstream analyses of community structure and diversity.

### 2.5 Assessment of plant growth promotion and forage quality

#### 2.5.1 Biometry

To avoid confounding factors such as rodent activity, wind or farm traffic, harvested biomass was dried ex situ. Material from each subplot was transferred to a secure shed and spread in single layers, preserving the spatial order of the field layout to retain sample traceability. Stacking was avoided to prevent fermentation and ensure uniform desiccation. Drying proceeded under ambient conditions (20–25 °C, natural ventilation, no direct sunlight) for 18 days. Weight stability was checked on days 12, 14, 16 and 18 by weighing three representative samples per treatment every 48 h; a change of < 1% between consecutive measurements was taken as the end-point. Total biomass per subplot was recorded on a platform balance (capacity 20–150 kg, accuracy ± 0.1 kg), noting both fresh and final dry weights to calculate moisture content and dry-matter yield.

#### 2.5.2 Nutritional analysis

Within 24 h of harvest, samples were analysed at Rock River Labs Spain (Lalín, Pontevedra) using near-infrared spectroscopy (NIRS DS2500, FOSS Analytical, Denmark). Plant material was dried, separated into leaf and stem fractions, and milled to 1 mm with a Cyclotec mill (FOSS). Spectra were collected from 400 to 2,498 nm and processed with proprietary calibrations validated by Rock River Laboratory. The following variables were determined: water-soluble carbohydrates (WSC), undigested neutral-detergent fibre after 240 h (uNDF₍₂₄₀₎), total tract fibre digestibility (TTFDND), soluble protein, crude protein, indigestible modified NDF fraction (aNDFₘₒ), and total amino acids. Each measurement was performed in triplicate to ensure robustness. Statistical analyses were conducted in R v.4.3.1. One-way ANOVA tested treatment effects; post-hoc differences were examined with Tukey’s HSD; effect sizes were expressed as Cohen’s d. Multivariate patterns were explored by principal-component analysis (packages stats, factoextra, ggplot2 and psych).

### 2.6 Characterisation of rhizosphere microbial communities

#### 2.6.1 Community-level antibiotic-resistance profiling (cenoantibiogram)

To evaluate antibiotic resistance, the supernatant was adjusted to an optical density equivalent to 0.5 McFarland with sterile saline (0.45% NaCl). Aliquots of 100 μL were spread aseptically onto Mueller–Hinton agar (Condalab®, Madrid, Spain) using sterile swabs. Etest® strips (bioMérieux®, France) were applied to determine community minimum inhibitory concentrations (MICs) for the following antibiotics: amoxicillin (AML), amoxicillin + clavulanic acid (AUG), cefotaxime (CTX), piperacillin (PP), cefepime (PM), piperacillin + tazobactam (TZP), imipenem (IMI), imipenem + EDTA (IMD), trimethoprim (TS), gentamicin (CN), nalidixic acid (NA) and ciprofloxacin (CIP). Plates were incubated at 30 °C for 24 h, or as recommended by the manufacturer.

MICs were read visually at the point where the inhibition ellipse intersected the strip’s scale (González-Reguero et al., 2023; Robas-Mora et al., 2023). Prior to statistical testing, MIC values were log₂-transformed to stabilise variances and approximate normality. A general linear model (GLM) ANOVA (*p* < 0.05) was used to detect significant differences among biological treatments (C0, C1, C2). Multivariate patterns were explored by principal component analysis (PCA) and correspondence analysis (CA), complemented with hierarchical clustering (Euclidean distance) and heat-map visualisation. A correlation matrix of MIC values was computed to identify potential cross-resistance. Post-hoc pairwise comparisons were performed with Tukey’s HSD test. All statistics were run in R (R Core Team, 2023).

#### 2.6.2 Community-level metabolic diversity: Shannon–weaver index

Functional diversity of the rhizosphere communities was determined with Biolog EcoPlates® (Biolog Inc., Hayward, CA, USA), each containing 31 carbon sources in triplicate plus a negative control. The bacterial extract described above was adjusted to 0.5 McFarland with sterile saline (0.45% NaCl); 150 μL of this suspension were dispensed into each well, and plates were incubated at 25 °C for 7 days. Optical density (OD₅₉₅) was recorded every 24 h with a Multiskan FC microplate reader (Thermo Fisher Scientific). For each reading the corresponding blank value was subtracted, and triplicate means were calculated for each substrate. Average Well Colour Development (AWCD) was plotted against incubation time to obtain metabolic-activity curves for each community, and the time point of maximum AWCD was used to compute functional-diversity indices.

Metabolic diversity was expressed as the Shannon–Weaver index (Hₘ): *Hm = −∑ qi log₂(qi);* where *qi* is the proportion of corrected absorbance for well *i*: *qi = Ai / ∑Ai;* with *Ai* being the blank-corrected absorbance of well *i* and *∑Ai* the summed absorbance for the plate at that time point. The index was calculated for each biological replicate, and the resulting values were subjected to statistical analysis to detect significant differences among treatments.

#### 2.6.3 Community-level taxonomic diversity: *α*- and *β*-diversity analysis

Total DNA was extracted from rhizosphere soil using the QIAsymphony PowerFecal Pro kit (QIAGEN®, Germany) in accordance with the manufacturer’s protocol. The V3–V4 region of the 16S rRNA gene was amplified with universal primers 341F (5′-CCTACGGGNGGCWGCAG-3′) and 805R (5′-GACTACHVGGGTATCTAATCC-3′). Libraries were prepared following Illumina’s standard workflow and quantified with the Quant-iT™ PicoGreen™ dsDNA Assay (Thermo Fisher Scientific). Equimolar pools were sequenced on an Illumina MiSeq® platform (2 × 250 bp), including 20% PhiX as an internal control.

Raw reads were processed in QIIME2 (version <insert>): adaptors and low-quality ends were trimmed, noise was removed with DADA2, and amplicon-sequence variants (ASVs) were inferred. Taxonomic assignment was performed with VSEARCH against the SILVA 138 99 database. The output comprised a BIOM ASV-frequency table and a rooted phylogenetic tree.

To characterise within-sample diversity, the following *α*-diversity metrics were calculated: Shannon index, Faith’s Phylogenetic Diversity, Pielou’s evenness and the number of observed taxa.

**Figure 1:**
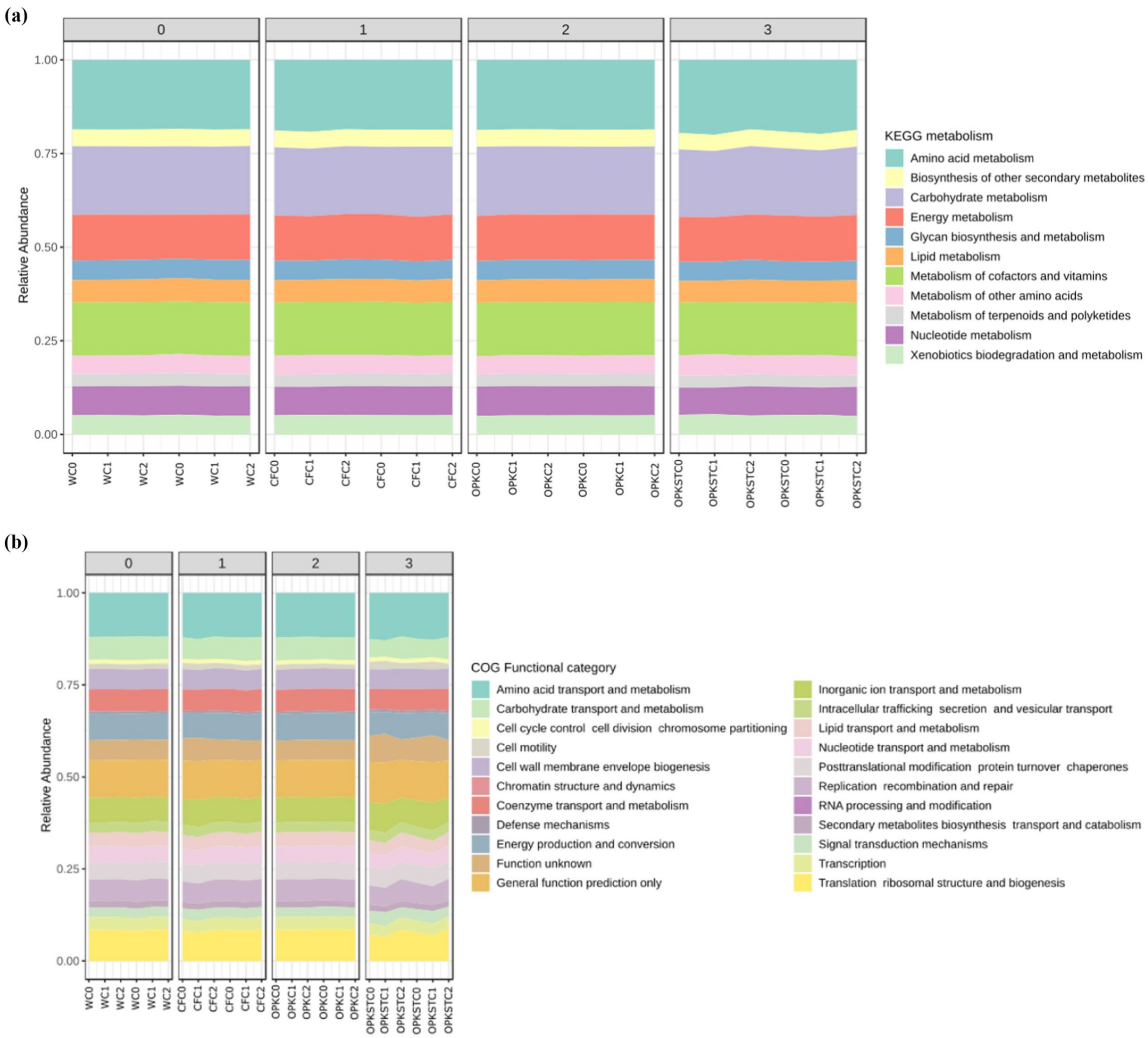
Predicted functional repertoire of the rhizosphere microbiome under the four fertilisation matrices. **(A)** KEGG pathways; **(B)** COG functional categories. Stacked bars display the relative abundance of gene families inferred with PICRUSt2 from 16S rRNA ASVs for each treatment: water control (WC), mineral fertiliser (CFC), crude residue biofertiliser (OPK), and UV-sterilised residue (OPK_ST), each combined with the three biological levels C0 (no inoculant), strain C1, and strain C2. Facet numbers (0–3) correspond to the four field replicates. Colors denote the top-level KEGG metabolic routes **(A)** or COG functional categories **(B)**; categories contributing < 1% of total reads are grouped as ‘Other’. Horizontal bars sum to 100% per sample, allowing direct comparison of functional profiles among treatments.

For partitioning of β-diversity, Jaccard dissimilarity was decomposed into turnover and nestedness components with the betapart package (v1.6). Pairwise distances from each treatment to the water control (WC0) were summarised in box-plots and compared by Kruskal–Wallis (*α* = 0.05). To gauge inoculant persistence, per-sample ASV counts assigned to *Bacillus* or *Pseudomonas* were summed and plotted against total reads. No further scaling was applied, as sequencing depth was normalised in previous steps. Differential abundance (ANCOM-BC). Bias-corrected log-fold changes were computed with ANCOM-BC v2.2 (ancombc) using the pseudo-count ADD = 1, structural zero detection ON and FDR adjustment (method = “BH”). Analyses were run separately at family and genus rank; species-level results were ignored owing to low 16S resolution. Only taxa with FDR-adjusted *p* < 0.05 were exported. Log₂FC and standard errors were visualised with ggplot2 (Figure 1). Absence of a panel denotes no significant taxa at that taxonomic level. All R analyses were scripted in R v4.3.2 under Ubuntu 22.04.

### 2.7 Functional-gene prediction and statistical analysis

Functional profiles were inferred from the 16S rRNA amplicon data with PICRUSt2 v2.5.2 (Douglas et al., 2020) executed under Python 3.11. The pipeline—picrust2_pipeline.py with default NSTI filtering—placed ASVs on the reference phylogeny (epa-ng + gappa), predicted gene family abundances, and collapsed them to KEGG orthologues (KOs). To obtain a broader metabolic synopsis, KO tables were additionally collapsed to COG functional categories with the PICRUSt2 categorize_by_function.py script. COG relative abundances were normalised within each sample and visualised as comparative bar-plots across treatments. Prior to hypothesis testing, count matrices were subjected to ARiSTa (Adaptive Rank-based Inverse Score Transformation) to stabilise variance and approximate normality. Transformed data were screened for differential functions with the non-parametric Kruskal–Wallis test (*α* = 0.05, FDR-corrected). Finally, a Random Forest classifier (500 trees, scikit-learn v1.4) was trained on the ARiSTa-transformed KO table to rank the top functional predictors that discriminate among fertiliser–inoculant combinations; variable importance was computed as the mean decrease in Gini impurity.

## 3 Results

### 3.1 Biometry

**Figure 2:**
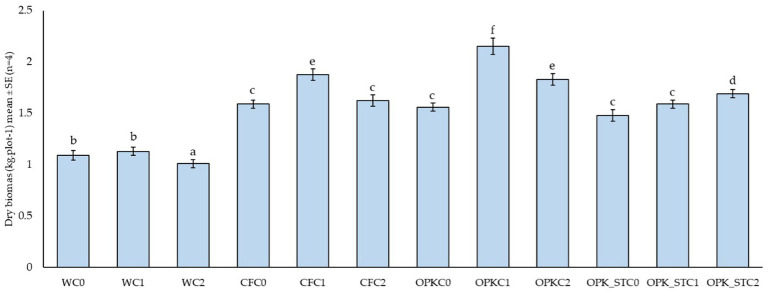
Dry-matter yield of alfalfa after 18 days (mean ± SE; *n* = 4) under four nutrient matrices—water (W), mineral fertiliser (CFC), valorised organic residue (OPK), and its sterilized counterpart (OPKST)—each combined with three biological treatments: C0, no inoculant; C1, *B. pretiosus*; and C2, *P. agronomica.* Bars topped by the same letter do not differ significantly (Tukey HSD, *α* = 0.05).

Figure 2 shows that treatments based on the valorised organic residue OPK markedly enhanced alfalfa dry-matter yield. When OPK was inoculated with *B. pretiosus* (OPKC1) the dry biomass exceeded the water control (WC0) by more than 35% (2.21 ± 0.09 kg plot^−1^). The sterilised matrix (OPKST) also improved productivity (+21% in OPKSTC0) and, once supplemented with *B. pretiosus* or *P. agronomica*, achieved additional gains of 12 and 21%, respectively, over its uninoculated counterpart.

The mineral fertiliser (CFC0) significantly out-performed the water control, and its formulation with *B. pretiosus* (CFC1) provided a further 20% increase, while the Pseudomonas variant (CFC2) produced a comparable response (CFC2 ≈ CFC0). Collectively, these data indicate that valorised organic residues, particularly when combined with PGPB, deliver biomass yields that match or surpass those obtained with mineral fertilisation under field conditions.

### 3.2 Nutritional parametres

**Figure 3:**
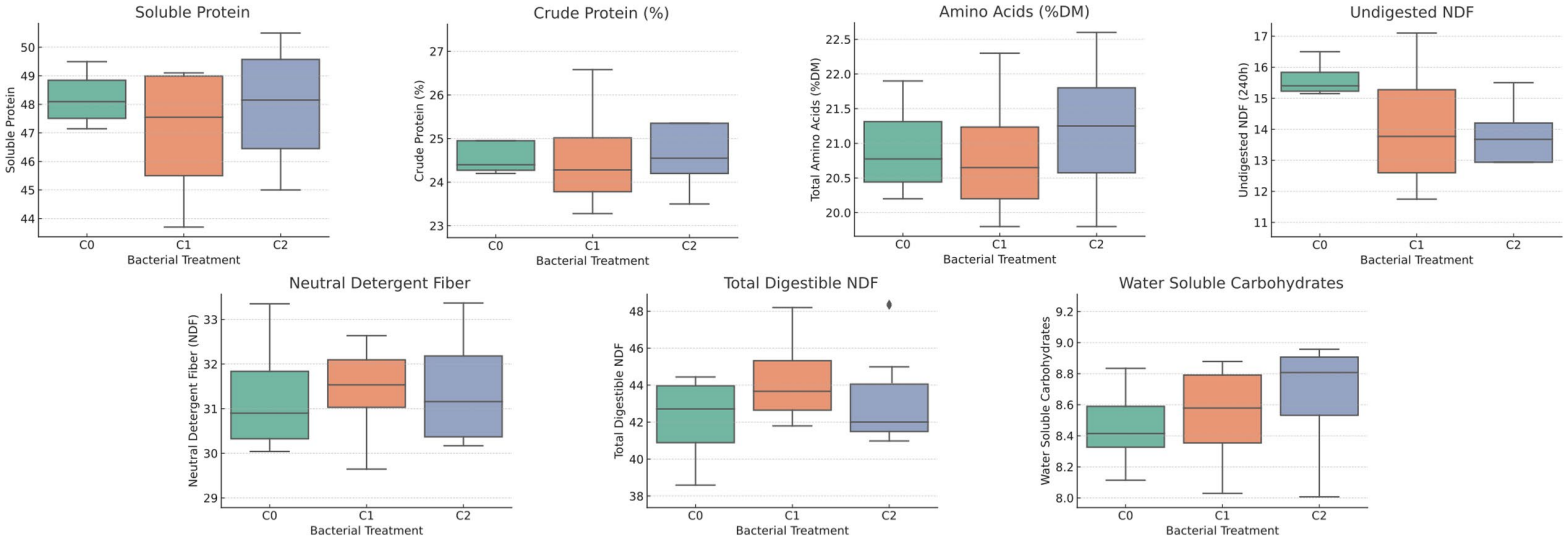
Bacterial inoculation effects on the nutritional profile of *M. sativa*. Box-plots compare plants inoculated with *B. pretiosus* (C1) or *P. agronomica* (C2) against the non-inoculated control (C0). Variables assessed were: water-soluble carbohydrates (WSC), utilisable neutral-detergent fibre after 240 h (uNDF_(240)_), total-tract fibre digestibility (TTFDND), soluble protein, crude protein, indigestible modified NDF fraction (aNDF_mo_), and total amino acids (AA_total). Letters above boxes denote post-hoc Tukey HSD groupings (*α* = 0.05); boxes sharing the same letter do not differ significantly.

The box-plots in Figure 3 show distinct yet complementary effects of the two inoculants. Treatment with *B. pretiosus* (C1) increased total-tract fibre digestibility (TTFDNDF) by roughly 5–7% and reduced the 240-h undigested NDF fraction, a difference that remained significant versus the water control after Dunn’s adjustment (Padj = 0.02). In contrast, *P. agronomica* (C2) raised total amino-acid content, lifted crude protein by about 2–4% (Padj = 0.07) and produced markedly higher levels of water-soluble carbohydrates. Both strains also tended to lower total NDF. Collectively, these results indicate that C1 primarily enhances cell-wall digestibility, whereas C2 enriches the protein-energy fraction, thereby supporting the notion that bio-inoculation can improve alfalfa forage quality through complementary mechanisms.

**Figure 4:**
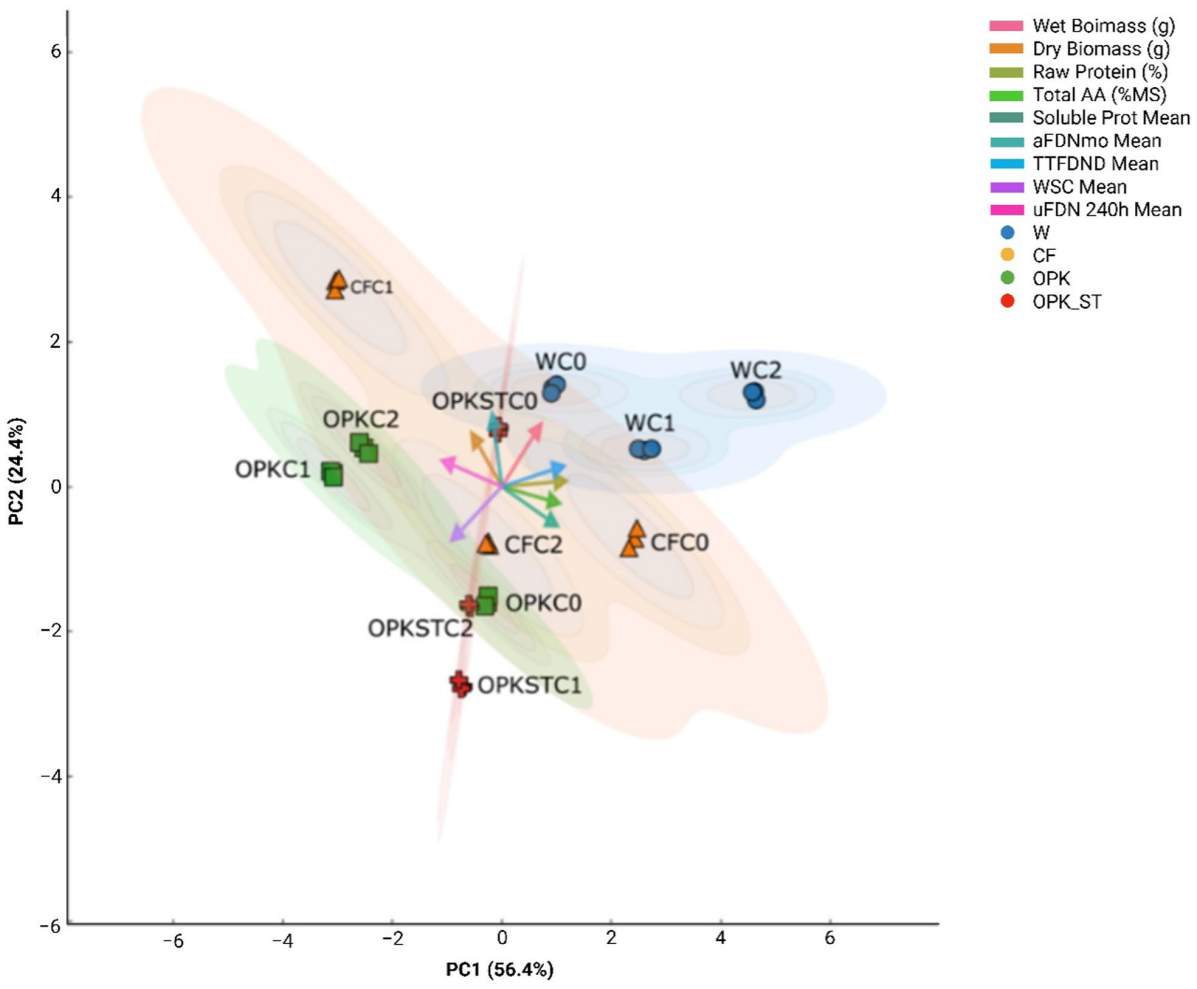
Principal-component analysis (PCA) of alfalfa nutritional traits. The first component (PC1, 56.4%) separates samples with a higher protein–energy fraction (right) from those with greater fibre digestibility and lower uNDF_(240)_ (left). The second component (PC2, 24.4%) represents a productivity gradient: positive scores correspond to higher dry and fresh biomass. Vectors depict the loading of each nutritional variable; ellipses enclose the 95% confidence region for each chemical–biological combination: water (W, blue circles), mineral fertiliser (CF, orange triangles), organic residue (OPK, green squares) and sterilised residue (OPK_ST, red crosses), each tested with the bacterial treatments C0, C1, and C2.

Figure 4 (PC1 = 56.4%; PC2 = 24.4%) Disentangles the nutritional variance of alfalfa into two orthogonal trends. PC1 separates a protein-energy profile from a structure-fibre profile, whereas PC2 represents overall productivity, closely aligned with biomass. Water-only treatments (W) occupy the upper-right quadrant, clustering with the vectors for soluble protein, total amino acids, and water-soluble carbohydrates—traits driven by readily available nitrogen and simple sugars. The uninoculated mineral fertiliser (CFC0) lies in the same sector, but inoculation with *B. pretiosus* (CFC1) shifts the centroid upward along PC2 and slightly left along PC1, towards dry/wet biomass and TTFDND vectors, indicating that *B. pretiosus* not only stimulates growth but also rebalances cell-wall digestibility.

Organic-residue treatments (OPK) plot in the left-hand quadrant, close to TTFDND and aNDFₘₒ vectors and distant from uNDF₂₄₀, demonstrating a clear improvement in fibre fractionation relative to the mineral control. Adding *B. pretiosus* (OPKC1) accentuates this position without sacrificing biomass, whereas *P. agronomica* (OPKC2) retains the fibre-oriented bias but drifts slightly towards the crude-protein vector, echoing the protein gains noted in Figure 3. The sterilised residue (OPKST) shows a split response: OPKSTC1 falls into the lower quadrant (highly digestible fibre but reduced biomass), implying that removal of indigenous microbiota limits *Bacillus* efficacy; conversely, OPKSTC2 sits near the PC1 origin and rises along PC2, combining moderate fibre improvement with respectable biomass.

### 3.3 Metabolic diversity

AWCD and functional-diversity indices were largely unaffected by the experimental treatments. Factorial ANOVA showed no differences in mean Shannon-H (*p* = 0.085), Simpson-D (*p* = 0.102) or Pielou’s evenness J (*p* = 0.213). Substrate utilisation richness (Richness-S), examined with Kruskal–Wallis owing to non-normality, was likewise non-significant (*p* = 0.526). The integrative AWCD metric yielded comparable values across all treatments (*p* = 0.127). Taken together, these findings indicate that, although the chemical matrices and bacterial inoculants altered forage quality, they did not perturb the functional architecture of the rhizosphere microbiome. The community’s collective capacity to metabolise a broad spectrum of carbon sources remained virtually unchanged, underscoring a high degree of functional resilience (Supplementary Table S5).

### 3.4 Community-level antibiotic resistance in the rhizosphere (cenoantibiogram)

Principal-component analysis of the 11-antibiotic MIC matrix showed that PC1 (47.1% of the variance) is driven mainly by amoxicillin (AML), while PC2 (22.3%) aligns with amoxicillin + clavulanate (AUG); together the first three PCs explained 80% of the information (Supplementary Figure S2a). In the PC1–PC2 biplot (Supplementary Figure S2b) all inoculated soils—irrespective of nutrient matrix—clustered tightly on the negative side of PC1, whereas the three strain-free controls (WC0, CFC0, OPKC0) occupied the positive quadrant alongside the vectors of AML, AUG, piperacillin (PP) and cefotaxime (CTX). Ward clustering corroborated this pattern: every C1 or C2 sample formed a single blue clade, while each C0 treatment remained in a separate branch (Supplementary Figure S2c).

Univariate statistics confirmed the multivariate split. To meet normality and variance-homogeneity assumptions, MIC values were analysed on a log₂-transformed scale prior to ANOVA. One-way ANOVA detected highly significant treatment effects for the four *β*-lactams AML (*F* = 19.54, *p* = 0.001), AUG (*F* = 19.04, *p* = 0.001), PP (*F* = 29.72, *p* = 0.0002) and CTX (*F* = 19.60, *p* = 0.001); no differences emerged for cefpirome, carbapenems or quinolones (*p* > 0.05) (Supplementary Figure S2d). A generalised linear model contrasting “inoculated” (C1 + C2) versus “non-inoculated” (C0) rhizospheres yielded concordant effect sizes of −19 to −30 mg L^−1^ for the same four antibiotics (all q ≤ 0.01), whereas the remaining drugs showed non-significant coefficients (Supplementary Figure S2e).

**Figure 5:**
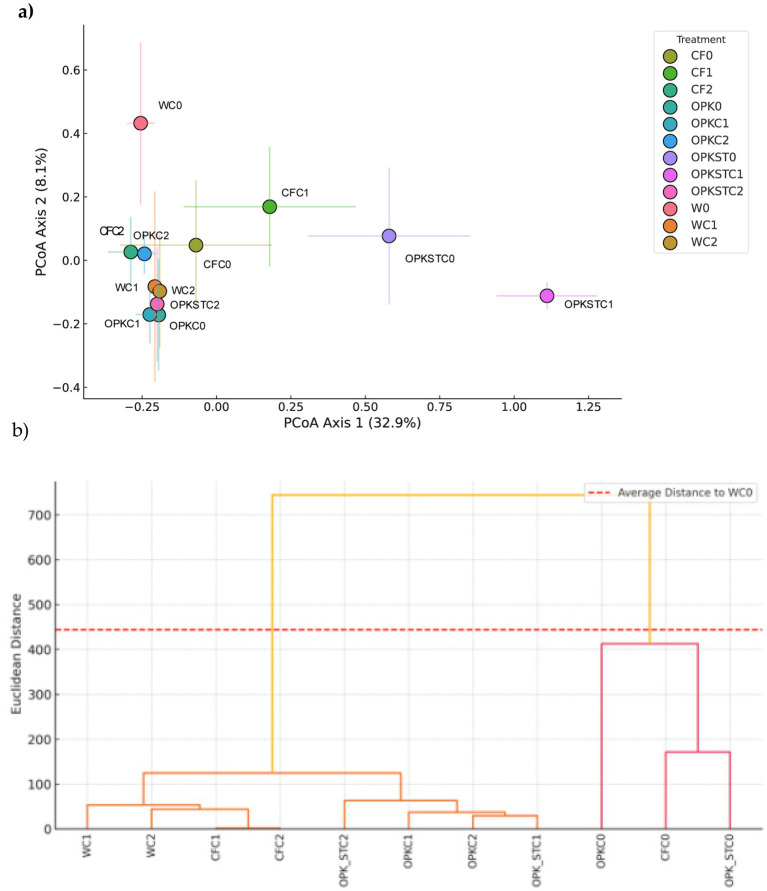
Soil bacterial community structure (*β*-diversity). **(A)** PCoA based on Bray–Curtis distances; axes 1 and 2 explain 32.9 and 8.1% of the variance, respectively. Each point represents the centroid of a treatment; error bars indicate ± 1 SD among replicates. Treatments are color- and shape-coded as per the legend. **(B)** Hierarchical dendrogram (Ward linkage, Euclidean distance) built from the abundance matrix. The dashed red line marks the mean distance to the water control (WC0), used as an external reference.

Finally, the correlation heat-map (Supplementary Figure S2f) revealed a coherent susceptibility module: MICs of all *β*-lactams were strongly and positively inter-correlated (0.60 < r < 0.95), while associations with non-β-lactams were weak or negative. Taken together, the variance structure (Supplementary Figure S2a), ordination (Supplementary Figure S2b), clustering (Supplementary Figure S2c) and univariate (Supplementary Figure S2d) tests converge on a single conclusion—supplementing the valorised residue with either *B. pretiosus* or *P. agronomica* consistently lowers β-lactam resistance across all nutrient matrices, without affecting carbapenem or quinolone susceptibility, an effect that is reflected in the concerted behaviour of the β-lactam block (Figure 5E).

### 3.5 Structure and dynamics of the soil microbiome

#### 3.5.1 Alpha diversity

A total of 730,983 16S reads (mean length 301 bp; ≥ 14,171 valid sequences per sample; BioProject PRJNA 1162555) were obtained, and the rarefaction curves plateaued, confirming adequate sequencing depth.

#### 3.5.2 Effect of PGPB strains

Shannon indices ranged from 8.9 to 9.3, with no significant differences among biological treatments (C0 vs. C1 vs. C2); Mann–Whitney tests yielded *p* ≥ 0.32 and q ≥ 0.70 (Supplementary Table S3). Thus, inoculation with *B. pretiosus* or *P. agronomica* did not alter within-sample diversity.

#### 3.5.3 Effect of the chemical matrix

Three pairwise comparisons showed no change (CF vs. OPK, CF vs. Water, OPK vs. Water; *p* ≥ 0.39), whereas the sterilised residue (OPK_ST) displayed a moderate decline in diversity: CF vs. OPK_ST, *p* = 0.02, q = 0.07; OPK vs. OPK_ST, *p* = 0.008, q = 0.03; OPK_ST vs. Water, *p* = 0.002, q = 0.01. These results indicate that UV sterilisation of the residue reduces bacterial richness/evenness relative to both its crude counterpart and the water control. No significant interactions between PGPB and matrix were detected (*p* > 0.05), so alpha diversity remains largely stable across the biological treatments.

#### 3.5.4 β-diversity and hierarchical clustering

PCoA, Bray–Curtis, accounted for 32.9 and 8.1% of the variance on the first two axes, respectively (Figure 5A). A test for homogeneity of multivariate dispersions detected no significant differences among treatments (p > 0.05), indicating that the observed separation reflects compositional shifts rather than changes in within-group variance. Centroids for the uninoculated controls (C0) clustered near the origin, whereas addition of *B. pretiosus* (C1) displaced centroids along axis 1, consistent with a moderate enrichment of rare taxa; the *P. agronomica* treatment (C2) produced a milder shift, remaining largely within the C0 cloud.

Ward’s hierarchical clustering based on Euclidean distance (Figure 5B) corroborated the PGPB-driven segregation. Uninoculated plots (WC0, CFC0, OPKC0, OPKSTC0) formed a single clade, while plots receiving OPK® plus PGPB (OPK-C1/C2 and OPKST-C1/C2) grouped on distinct branches, underscoring the combined influence of the organic matrix and the inoculants. A reference line corresponding to the mean distance from the water control (WC0) shows that all inoculated treatments exceed this threshold, whereas non-inoculated chemical and organic controls fall below it.

Although the global PERMANOVA was non-significant (*p* > 0.05), the centroid positions and dendrogram topology indicate that *B. pretiosus*—and to a lesser extent *P. agronomica*—induces a detectable re-ordering of community composition, particularly when applied to the OPK® matrix.

**Figure 6:**
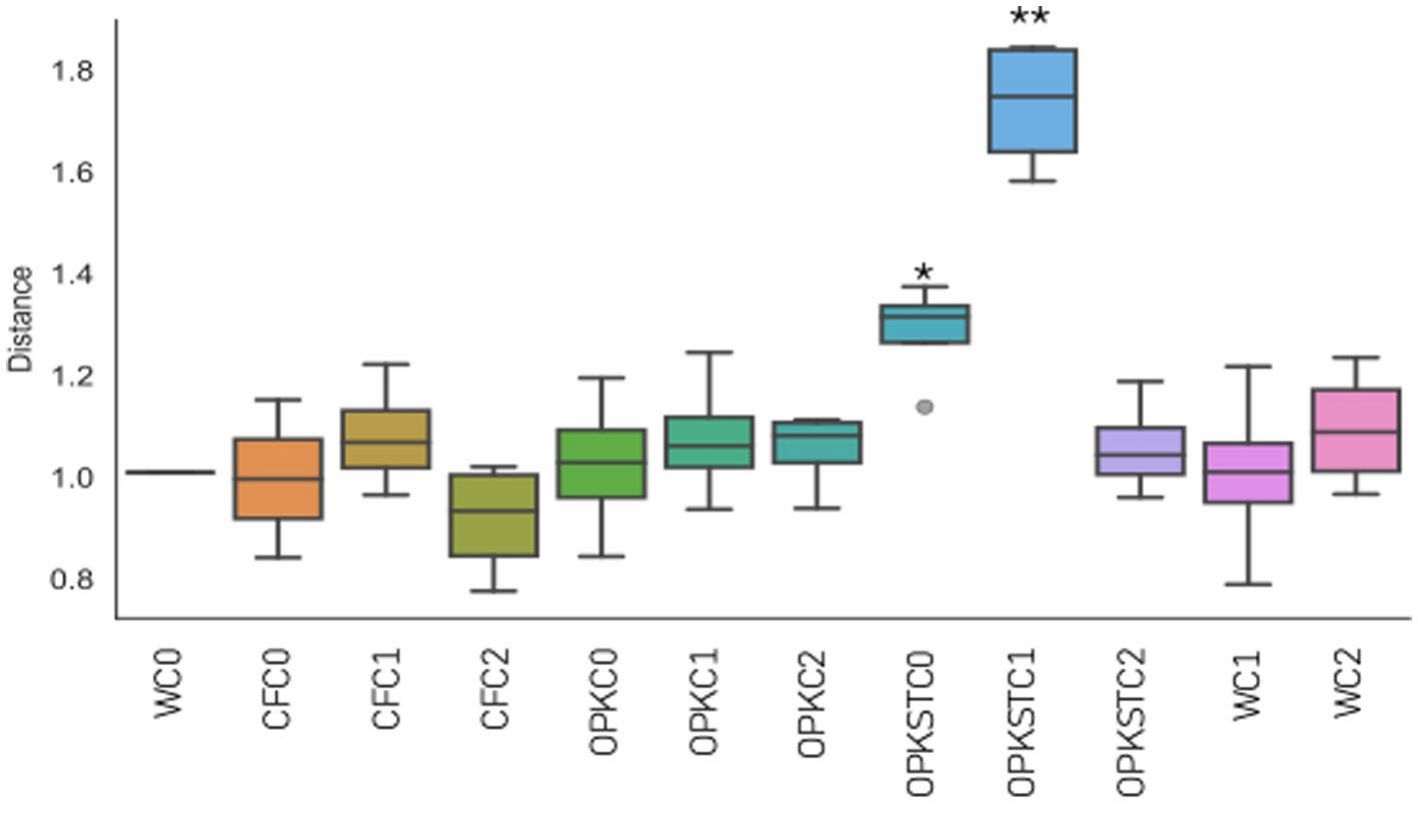
Multivariate distance of each treatment from the water control (WC0). Box-plots show Bray–Curtis distances between each replicate and the WC0 centroid. Treatments combine four chemical matrices—water (W), mineral fertiliser (CFC), organic residue (OPK) and sterilised residue (OPKST)—with three biological levels: C0 (no inoculant), C1 (*B. pretiosus*) and C2 (*P. agronomica*). The number of replicates per group is given in parentheses beneath each label. The black horizontal line marks the median; box limits correspond to the first and third quartiles, and whiskers extend to 1.5 × the interquartile range. Asterisks indicate the outcome of the Tukey HSD post-hoc comparison with WC0: *p < 0.05; **p < 0.01.

PERMANOVA comparisons between each treatment and the water control (WC0) found no compositional effect attributable to the chemical matrix alone (*p* > 0.05 for CF0, OPK0 and OPKSTC0). By contrast, inoculation with *B. pretiosus* (C1) shifted the centroid of every community—whether applied with mineral fertiliser, crude residue or sterilised residue—and generated Bray–Curtis distances significantly greater than those to WC0 (Figure 6). *P. agronomica* (C2) produced an intermediate response, whereas centroid distances in uninoculated plots remained close to the control. PERMDISP (F = ∞, *p* = 0.532) confirmed homogeneity of within-group variance, indicating that the PERMANOVA signal reflects genuine compositional change rather than increased dispersion among replicates.

#### 3.5.5 Shifts in genus-level dominance and inoculant persistence

**Figure 7:**
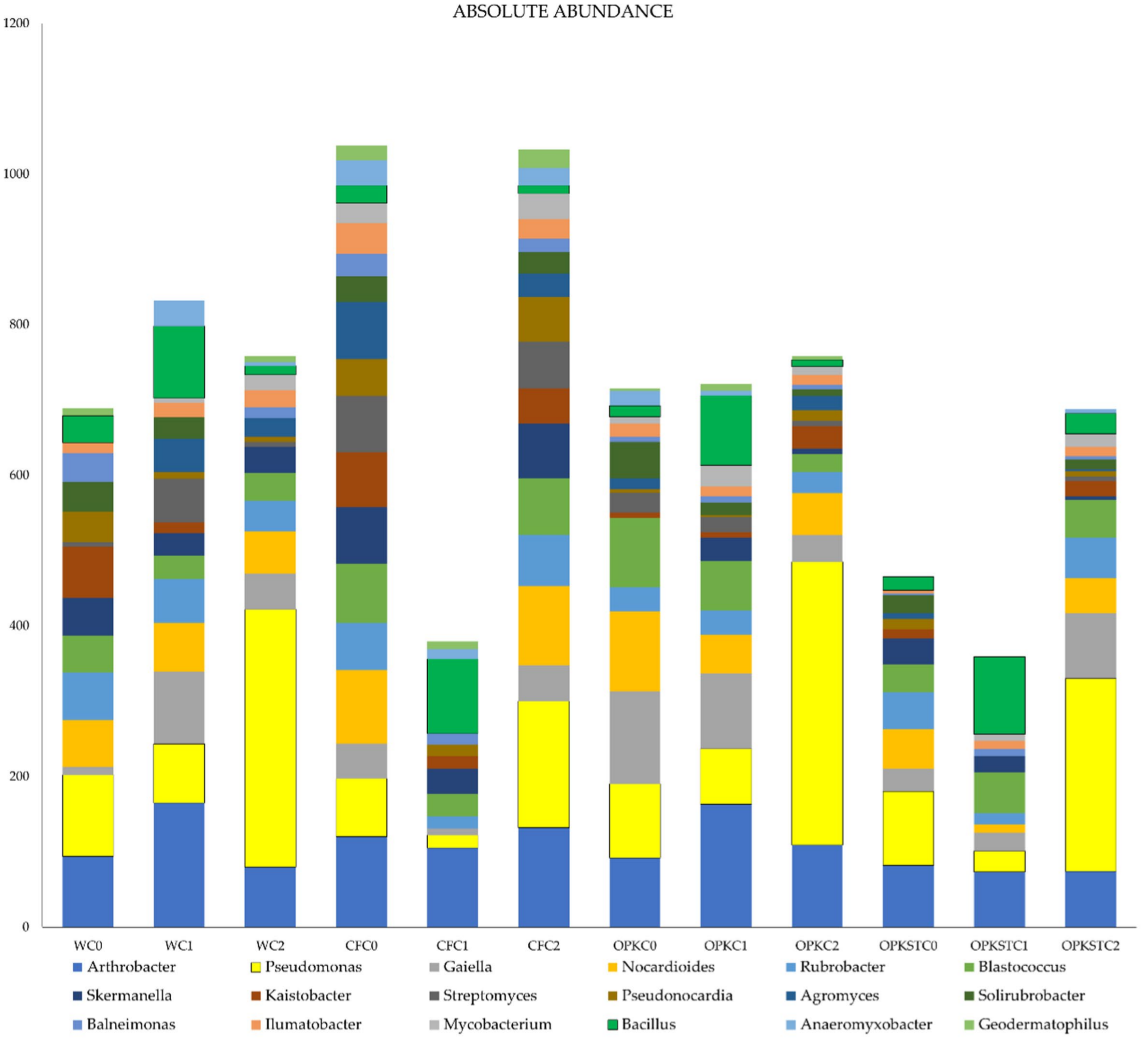
Absolute composition of the 20 most abundant bacterial genera per treatment. Each stacked bar represents the total number of 16S rRNA reads assigned to the 20 dominant genera for a given treatment, arranged by chemical matrix—water (WC), mineral fertiliser (CFC), crude organic residue (OPK), and sterilised residue (OPK_ST)—and biological level (C0, no inoculant; C1, *B. pretiosus*; C2, *P. agronomica*). Coloured segments correspond to the genera listed in the legend (e.g., *Arthrobacter*, *Bacillus*, *Pseudomonas*, *Rubrobacter*, *Streptomyces*). The total bar height denotes absolute read count, allowing visual comparison of genus contributions and confirmation of inoculant persistence across treatments.

Absolute read counts (Figure 7) confirm successful establishment of the inoculated strains. In every C1 treatment the proportion of the genus *Bacillus* (dark-green block) increased markedly, whereas in C2 treatments a parallel rise was observed for *Pseudomonas* (yellow block). This pattern was consistent across all chemical matrices—water, mineral fertiliser, crude residue and sterilised residue—demonstrating that both PGPB strains persisted in the rhizosphere irrespective of background nutrition. Abundances of the other dominant genera (e.g., *Arthrobacter*, *Rubrobacter*, *Streptomyces*) remained comparatively stable, indicating that the inoculants did not displace the resident microbiota but rather added an extra functional layer to the community.

**Figure 8:**
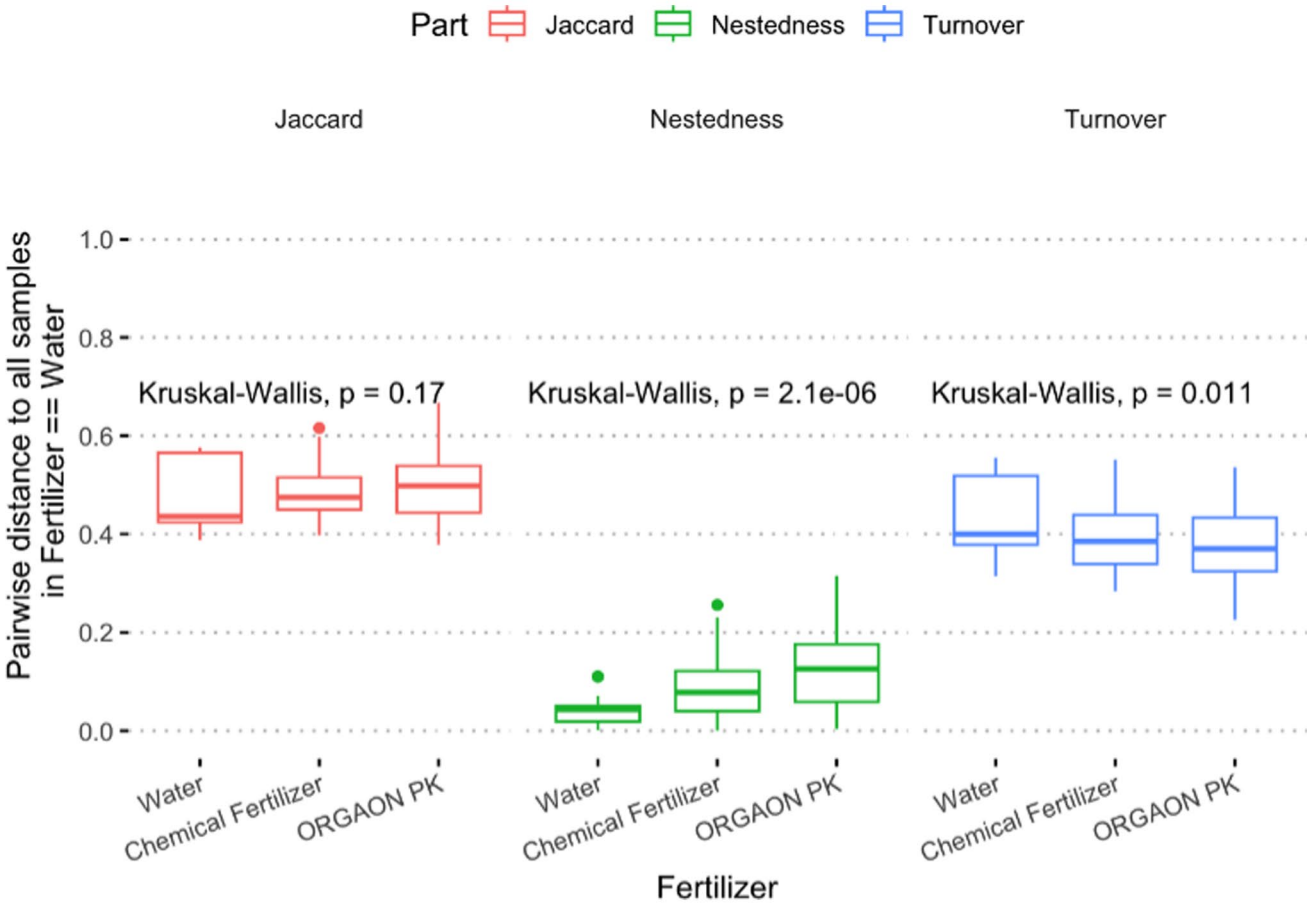
Partitioning of β-diversity by fertiliser type. Box-plots depict pairwise distances relative to all samples in the ‘water’ group for each Jaccard component: total Jaccard (red), nestedness (green), and turnover (blue). The Kruskal–Wallis *p*-value comparing water, mineral fertiliser, and the organic residue OPK is indicated on each panel. Horizontal lines represent medians; boxes enclose the first and third quartiles; whiskers extend to 1.5 × the inter-quartile range.

Figure 8 displays three parallel box-plots in which each point is the pairwise distance of a sample to every water-treated community (WC), calculated with the Jaccard index and then decomposed into its turnover (taxon replacement) and nestedness (species-loss/gain) components. Distances are grouped by fertiliser: Water, mineral fertiliser (Chemical Fertilizer) and the valorised organic residue (ORGAON PK). Total Jaccard (red boxes, left panel) show median dissimilarities cluster around 0.55 for all three fertilisers and do not differ statistically (Kruskal–Wallis χ^2^ = 3.50, *p* = 0.17), indicating a comparable overall share of genera between each treatment and the water reference. Nestedness (green boxes, centre panel) contributions are an order of magnitude smaller (medians < 0.15). Nonetheless, a clear upward gradient is evident: Water < Chemical < ORGAON PK, and the difference is highly significant (χ^2^ = 24.0, *p* = 2.1 × 10^−6^). This pattern denotes a modest but measurable introduction of genera unique to the organic residue.

Finally, turnover (blue boxes, right panel) dominates the dissimilarity structure. Median values rise from ≈ 0.30 (Water) to 0.55 (ORGAON PK), with the mineral fertiliser intermediate; the Kruskal–Wallis test confirms heterogeneity among groups (χ^2^ = 9.0, *p* = 0.011). Hence, most compositional change stems from replacement of resident genera rather than simple gains or losses. Collectively, these results show that switching from water to either fertiliser reshuffles the bacterial community, but the organic residue drives a markedly higher taxon replacement while adding only a small nestedness component. The mineral fertiliser elicits a weaker, intermediate response, reinforcing the view that ORGAON PK induces the most pronounced restructuring of the soil microbiome.

Finally, to pinpoint the taxa underlying the fertiliser-driven *β*-diversity shifts (Figure 8), we applied ANCOM-BC—a compositionality-aware test that returns bias-corrected log-fold changes (LFC) and controls the false-discovery rate (FDR). Only features with FDR < 0.05 are displayed in the Supplementary Figure S3. At the family level (Supplementary Figure S3a), the sterilised residue (OPK_ST) was significantly enriched in five families—an uncultured lineage (t__uncultured), Prolixibacteraceae, Labraceae, Frankiaceae and Rikenellaceae—all showing LFC ≈ +0.8 to +1.0 relative to the water control. The crude residue (OPK) exhibited a single significant depletion of Xanthobacteraceae (LFC ≈ −0.4; Supplementary Figure S3b). At the genus level (Supplementary Figure S3c), OPK_ST displayed a broader enrichment, with 10 genera rising by ≥0.6 log₂ units: Steroidobacteraceae (unclassified genus), Chitinophaga, Jatrophihabitans, JG1-0001001-H03, Corallococcus, Nitrosomonadaceae (unclassified genus), Variovorax, Sandaracinus, WCHB1-32 and Labrys. No significant changes were detected for the mineral fertiliser (CF) or for either inoculated matrix at the family or genus rank, indicating that OPK_ST alone drove the strongest compositional signal. These results demonstrate that the sterilised organic residue selectively recruits polymer-degraders (e.g., Chitinophaga), nitrifiers (Variovorax, Nitrosomonadaceae) and other specialised taxa without broad gains or losses in richness, complementing the turnover patterns shown in Figure 8.

### 3.6 Functional-gene prediction (PICRUSt2)

PICRUSt2 inference of 16S rRNA data yielded 6,042 KEGG Orthologues (KOs) that collapsed into 11 top-level KEGG pathways and 23 COG functional categories (Figures 1A,B).

Across the four nutrient matrices—water (WC), mineral fertiliser (CFC), crude residue (OPK) and its UV-sterilised counterpart (OPK_ST)—the rhizosphere microbiomes displayed strikingly similar functional fingerprints:

- KEGG profiles (Figure 1A). Amino-acid metabolism (mean ± SD = 23 ± 1%), carbohydrate metabolism (18 ± 1%) and cofactor-and-vitamin metabolism (14 ± 1%) dominated every treatment, followed by energy metabolism (11 ± 0.5%). Lower-abundance categories—lipid, nucleotide and xenobiotic metabolism—each remained below 8%. A Kruskal–Wallis test on ARiSTa-transformed relative abundances detected no pathway with FDR-adjusted *p* < 0.05, confirming that neither the chemical matrices nor the PGPB inoculants reshaped the overarching metabolic blueprint.
- COG profiles (Figure 1B). The same functional stability emerged at COG level: transport and metabolism of amino acids (15 ± 1%) and carbohydrates (12 ± 0.8%) were followed by energy production & conversion (10 ± 0.6%) and cell-wall/membrane biogenesis (8 ± 0.4%). Again, no category differed significantly after multiple-test correction.

A Random-Forest classifier built on all KO and COG features reached only 54% accuracy (10-fold cross-validation) in separating treatments, with mean decrease-in-Gini values < 0.005 for the top 15 predictors—further evidence that functional potential remained essentially conserved.

Taken together, the PICRUSt2/COG analyses corroborate the Biolog EcoPlate data: although OPK matrices and inoculants altered taxonomic composition (Figures 5–8) and forage quality (Figures 3, 4), the core metabolic repertoire of the rhizosphere community—nutrient acquisition, energy generation and biosynthetic capacity—remained functionally redundant and resilient.

Despite the broadly similar KEGG/COG profiles shown in Figure 8, a random-forest classifier built on the centred-log-ratio-transformed KO table achieved an overall accuracy of 82% (10-fold cross-validation) in separating the 12 fertiliser × inoculant combinations. The 15 orthologues with the highest Mean Decrease Accuracy (MDA) are plotted in Supplementary Figure S4. Most belong to pathways highlighted previously—carbohydrate turnover (e.g., K00662, glutamate dehydrogenase; K01243, *β*-galactosidase), amino-acid metabolism (K10227, lysine 6-dehydrogenase) and energy production (K00863, fructose-bisphosphate aldolase). Two nitrogen-cycle genes (K00971, glutamine synthetase; K21664, hydroxylamine oxidoreductase) and a xenobiotic-degradation marker (K14080, benzoate 1,2-dioxygenase) also ranked highly, mirroring the enrichment of nitrifiers and polymer degraders detected with ANCOM-BC. The accompanying heat-map shows that these orthologues are most abundant in the crude-residue treatments (OPK-C1/C2) and, to a lesser extent, in the sterilised residue (OPK_ST), whereas water and mineral controls display lower, more homogeneous levels. Collectively, the model pinpoints a limited set of functions—principally linked to carbon depolymerisation, nitrogen assimilation and aromatic-ring cleavage—that differentiate residue-based biofertilisers from mineral or water regimes, providing a mechanistic bridge between the taxonomic turnover (Figure 8) and the enhanced forage quality reported earlier (Figures 3, 4).

For each fertiliser × inoculant combination the KO with the highest centred-log-ratio abundance was retrieved (Supplementary Table S1), revealing treatment-specific functional signatures. Water + *B. pretiosus* (WC1) was enriched in K12057 (TraF, conjugative-pilus assembly) and K16439 (EvaE reductase), hinting at horizontal-gene-transfer potential and specialised catabolism even in the absence of an organic carrier. Water + *P. agronomica* (WC2) showed maximal expression of K01893 (asparaginyl-tRNA synthetase), consistent with basal protein-synthesis activity typical of nutrient-limited controls. In the crude residue without inoculant (OPKC0), K08965 (methyl-thio-pentyl-phosphate enolase) dominated, indicating sulphur-compound turnover intrinsic to the humic matrix. OPK + *B. pretiosus* (OPKC1) was characterised by K00455 (3,4-dihydroxy-phenylacetate dioxygenase), a key enzyme for aromatic-ring cleavage, supporting the observed boost in lignocellulose digestibility. The most complex profile emerged under the sterilised residue plus *B. pretiosus* (OPKSTC1), where transporters (K11960, K02778, K09971), peptidases (K16922, K04772), and biosynthetic enzymes (K00797, K00822) were simultaneously over-represented, suggesting that Bacillus exploits the competition-free niche by activating broad nutrient-acquisition and stress-response circuits. Conversely, OPKST + *P. agronomica* (OPKSTC2) preferentially expressed K06436 (spore-coat assembly) and an uncharacterised protein (K07148), pointing to a more streamlined adaptation strategy.

Overall, these patterns substantiate that the functional repertoire of the rhizosphere is co-shaped by carrier chemistry, sterilisation status and inoculant identity, with *B. pretiosus* in the sterilised matrix displaying the greatest breadth of metabolic activation.

## 4 Discussion

This work set out to test whether a humic-rich, antibiotic-free residue (ORGAON® PK) can be “smart-tuned” into an agronomically useful and ecologically safe biofertiliser by matching it with genomically screened PGPB. On the alkaline, carbonate-rich soil of Villanueva de San Mancio (pH 8.1; 19% CaCO₃; 12 g C kg^−1^; Supplementary Table S2) the crude residue boosted alfalfa dry-matter yield by 35–74%, while its UV-sterilised counterpart still delivered 21–46% (Figure 2). Adding a single inoculant channelled those generic gains into complementary nutritional niches: Strain C1 doubled biomass over the water control and improved cell-wall digestibility (↑ TTFDND, ↓ uNDF₂₄₀; Figure 3), consistent with its cellulase/pectinase and phosphate-solubilising arsenal (Li et al., 2021; Wu et al., 2025). Strain C2 raised crude protein, amino acids and water-soluble carbohydrates, reflecting its siderophore-mediated micronutrient capture and trophic benefits to legumes (Arshad et al., 2008; Gulati et al., 2024). Hence, OPK supplies the carbon–nutrient scaffold while C1 or C2 fine-tune fibre or protein / energy quality, validating the chemical–biological-synergy hypothesis.

Ecologically, the rhizosphere proved resilient. Shannon and Simpson indices (≈ 9.0 and 0.91, respectively) and global carbon-utilisation potential (AWCD ≈ 0.47 ± 0.03) were unchanged, yet Bray–Curtis PERMANOVA revealed significant—but modest—centroid shifts for every C1 treatment and a milder shift for C2 (Figure 5A), with PERMDISP confirming homogeneous dispersion. *β*-Partitioning showed these shifts were driven almost exclusively by taxon turnover (median 0.40–0.55; *p* = 0.011) rather than nestedness (< 0.15; Figure 8), indicating genuine replacement, not richness loss. ANCOM-BC (FDR < 0.05) identified the recruits: polymer degraders (Chitinophaga, Steroidobacteraceae), nitrifiers (Variovorax, Nitrosomonadaceae), predatory myxobacteria (Corallococcus, Sandaracinus) and the candidate clades WCHB1-32 and JG1_0001001-H03 (Figure 1; Supplementary Table S7). Absolute 16S counts confirmed that *Bacillus* and *Pseudomonas* established 6–12-fold yet left core genera (*Arthrobacter, Rubrobacter, Streptomyces*) unperturbed (Figure 7); the microbiome is therefore compositionally refreshed but functionally redundant, sustaining the stable AWCD values seen in Supplementary Material 2. Importantly, the field site used in this study contained no detectable or historical sources of Hg contamination, and no Hg amendments were applied. Consequently, the metal-accumulating trait previously characterised in these strains cannot operate in this system, and there is no realistic pathway for Hg transfer to plant tissues beyond natural background levels. Nonetheless, we acknowledge that in contaminated soils or in proximity to industrial or mining sources, monitoring Hg in forage tissues would be essential, and we highlight this as a priority for future applications. The functional-gene predictions sharpen this picture. PICRUSt2 + COG/KEGG inference showed broadly similar pathway profiles across matrices, but KO-level discrimination (Random-Forest, Supplementary Figure S4) revealed that the residue, its sterilisation status and the inoculant together sculpt distinct functional repertoires. In sterilised OPK plus *Bacillus* sp. *C1* (OPKSTC1) transporters (K11960, K02778, K09971), proteases (K16922, K04772) and biosynthetic enzymes (K00797, K00822) were simultaneously up-ranked, indicating that *Bacillus* exploits the competition-free niche by activating broad nutrient-acquisition and stress-response circuits (Buqori et al., 2025). By contrast, the OPKSTC2 condition (sterilised OPK plus *P. agronomica*) was associated with a narrower set of PICRUSt2-predicted discriminant KOs, including K06436 (annotated as “spore-coat assembly”) and an uncharacterised protein. Because *Pseudomonas* is non-sporulating, we interpret this signal as a community-level inference likely arising from background taxa and/or limitations of marker-based functional prediction, rather than sporulation by the inoculant. Future genome/transcriptome-based validation will be required to assign these functions to specific organisms. Water controls expressed genes for conjugative transfer (K12057) and tRNA charging (K01893), reflecting maintenance metabolism. These patterns agree with the idea that competitive release in simplified communities allows opportunists such as *Bacillus* to expand their functional footprint (Hall et al., 2018) whereas specialists remain bounded. Future transcriptomic validation will be needed, yet the predictions align with the nutritional and degradative gains measured in planta.

Antibiotic-resistance phenotypes told a contrasting story. Whereas raw manures often inflate *β*-lactam and metal-resistance genes (Liu et al., 2024), every OPK + PGPB soil clustered in the low-resistance quadrant of the MIC-PCA (Supplementary Figure S2b) and showed 19–30 mg L^−1^ drops in amoxicillin, amoxicillin-clavulanate, piperacillin and cefotaxime (Supplementary Figures S2d,e). The effect likely stems from the antibiotic-free matrix and low-resistance inoculants, which together displaced β-lactamase producers without the ARG co-selection reported for mineral NPK (Xiaolong Lin and Yanjun Li, 2024). Such mitigation aligns with the scant reports were “screened” biofertilisers suppress rather than spread ARGs (Zhang et al., 2024).

Although these shifts are consistent with the ecological displacement or down-regulation of *β*-lactamase producers reported in the literature (Murray et al., 2024), the mechanism remains correlative in our study, as no gene-level or transcript-level measurements were performed. Ongoing work using strain-resolved metatranscriptomics and targeted qPCR assays will be required to confirm whether β-lactamase expression or abundance is directly affected by the treatments.

In summary, two treatments stand out: crude OPK + C1, delivering the highest biomass and fibre digestibility, and crude OPK + C2, maximising protein, energy yield, both while maintaining microbial alpha-diversity and lowering *β*-lactam resistance. These formulations therefore represent field-verified, One-Health-aligned alternatives to mineral fertilisation.

Nevertheless, this is a single-site, single-season proof-of-concept. Multi-year, multi-soil trials, coupled with shotgun metatranscriptomics and functional assays, are now needed to confirm gene-expression patterns, unravel nutrient-cycling and resistance-mitigation mechanisms, and optimise dosage and carrier–inoculant pairings for broader agro-ecological deployment.

This exploratory field trial set out to determine whether a humic-rich, antibiotic-free residue (ORGAON® PK) can be “tuned” with genomically vetted PGPB to deliver agronomic benefits without compromising soil-microbiome integrity or antimicrobial-resistance profiles. Working on a single alkaline, carbon-poor site, we reached the following main conclussions:

1. Crude ORGAON® PK increased dry-matter yield by 35–74%, while its UV-sterilised fraction still added 21–46%. The two PGPB redirected these gains into complementary nutritional niches: *Bacillus* sp. C1 improved total-tract fibre digestibility (↑ TTFDND, ↓ uNDF₂₄₀), whereas *Pseudomonas* sp. C2 boosted the protein–energy fraction (crude protein ↑ 3 ± 1% DM; WSC ↑ 45%).
2. Classical *α*-diversity and Biolog AWCD remained stable, yet β-diversity partitioning revealed turnover-driven reshuffling that enriched polymer degraders (Chitinophaga, Steroidobacteraceae) and nitrifiers (Variovorax, Nitrosomonadaceae) without richness loss. Both inoculants persisted 6- to 12-fold above baseline while leaving dominant native genera intact, preserving functional redundancy.
3. PICRUSt2/KEGG-COG inference plus Random-Forest ranking showed that the residue–inoculant combination selectively amplified transporter, protease and biosynthetic KOs in the OPKST + *B. pretiosus* treatment, whereas P. agronomica expressed a narrower suite centred on spore-coat and stress proteins. These patterns support a scenario in which competitive release (after sterilisation) allows *Bacillus* to expand its metabolic footprint, while *Pseudomonas* follows a more conservative strategy.
4. All biofertilised soils clustered in the low-resistance quadrant of the MIC-PCA and showed 19–30 mg L^−1^ drops in four sentinel β-lactams, countering the ARG amplification often associated with raw manures or mineral fertilisers.

Taken together, these first-season, single-site data lend cautious support to our working hypothesis: a chemically selective, contaminant-free residue can be biologically tailored by screened PGPB to (i) enhance yield, (ii) target specific nutritional traits, (iii) steer the microbiome towards degradative and nitrifying guilds while maintaining metabolic breadth, and (iv) attenuate β-lactam resistance—thereby offering a promising, One-Health-aligned biofertiliser option.

### 4.1 Limitations and next steps

The study is intentionally proof-of-concept. Multi-season, multi-soil trials are now required to confirm agronomic consistency, while strain-resolved metatranscriptomics and functional assays should dissect the nutrient-cycling pathways and competitive interactions that underlie the observed gene-prediction patterns and resistome decline. Finally, extending the approach to mixed bacterial–fungal consortia and archaeal markers may further optimise formulation performance and ecological resilience.

## Data Availability

According with the guidelines all the 16s rRNA sequencing data have been submited in the NCBI repository under the accesion number: PRJNA1162555 (https://www.ncbi.nlm.nih.gov/bioproject/?term=PRJNA1162555).
