# Supplementary material for: Use of PGPB in bio-fertilisation: preserving the soil microbiome and enhancing field production of alfalfa
Source: Front Microbiol. 2026 Jan 20;16:1735729. doi: 10.3389/fmicb.2025.1735729 (PMC12864390; doi:10.3389/fmicb.2025.1735729)
Supplement: Supplementary file 1 [file Table_1.DOCX]

**Supplementary material**

***Table S1.*** *Physicochemical, elemental and microbiological characterisation of the organic fertiliser ORGAON® PK*

| HEAVY METALS (RD 506/2013) | | | | | |
| --- | --- | --- | --- | --- | --- |
| **Parameter** | **Result** | **Method** | **Technique** | |  |
| Cadmium (total) | 1.02 mg kg⁻¹ | LAB 1-02-12 | ICP-MS | |  |
| Copper | 3.8 mg kg⁻¹ | LAB 1-02-12 | |  |  |
| Nickel (total) | 2.55 mg kg⁻¹ | LAB 1-02-12 | |  |  |
| Lead (total) | 0.13 mg kg⁻¹ | LAB 1-02-12 | |  |  |
| Zinc (total) | 22.02 mg kg⁻¹ | LAB 1-02-12 | |  |  |
| Mercury (total) | <0.02 mg kg⁻¹ | LAB 1-02-12 | |  |  |
| Chromium (total) | 2.68 mg kg⁻¹ | LAB 1-02-12 | |  |  |
| Chromium VI | <0.200 mg kg⁻¹ | Subcontracted | Spectrophotometry | |  |
| MACRO‑/MICRO‑NUTRIENTS | | | | | |
| Total nitrogen | 0.5 % | LAB 1-03-70 | Volumetry | |  |
| Watersoluble P₂O₅ | 5.29 % | LAB 1-02-12 | ICP-MS | |  |
| Watersoluble K₂O | 7.84 % | LAB 1-02-12 | ICP-MS | |  |
| Total organic matter | 9.59 % | LAB 1-03-37 | Gravimetry | |  |
| Total organic carbon | 5.56 % | LAB 1-03-37 | Gravimetry | |  |
| pH (20ºC) | 5.1 | LAB 1-03-77 | Potentiometry | |  |
| Density (20ºC) | 1.18 g cm⁻³ | LAB 1-03-25 | Densimetry | |  |
| HUMIC FRACTIONS | | | | | |
| Humic acids | <0.75 % | LAB 1-03-45 |  | |  |
| Fulvic acids | 6.79 % | LAB 1-03-45 |  | |  |
| Total humic extract | 6.79% | LAB 1-03-45 |  | |  |
| MICROBIOLOGICAL SAFETY | | | | | |
| *Salmonella spp* | Not detected in 25 g | Subcontracted | Enrichment & plating | |  |
| *Escherichia coli* | < 3 MPN g⁻¹ | Subcontracted | Most‑probable‑number | |  |
| ADDITIONAL CONTAMINANT | | | | | |
| Furfural (2‑furfuraldehyde) | <0.03 % (w/w) | Subcontracted | HPLC-UV | |  |

All values correspond to the concentrate prior to 1 : 512 dilution for field application.

| ***Table S2.*** *Phenotypic, chemotaxonomic and genomic analysis of strains SAICEU11T (C1) and SAICEU22T (C2).* | | |
| --- | --- | --- |
| Phenotypic characterization | | |
|  | SAICEU11^T^ | SAICEU22^T^ |
| Cell size | 2±0.10 x 0.70±0.15 | 1.40±0.10 x 0.50±0.15 |
| Isolation source | *M. sativa* rhizosphere | *M. sativa* |
| Temperature range | 4-37 ºC | 5-40 ºC |
| OGT | 28 ºC | 28 ºC |
| pH range | 5.5 – 8.0 | 5.5 – 8.0 |
| pH | 7.0 | 7.0 |
| Pyoverdine | - | + |
| Gelatin | + | + |
| Nitrate | - | + |
| D-glucose | + | + |
| Lactose | + | - |
| D-Mannitol | - | + |
| Citrate | - | + |
| Maltose | - | + |
| Xylose | - | + |
| IAA (µg. mL10^-1^) | 5.61± 0.03 | 5.85± 0.09 |
| ACCd (p/a) | - | + |
| Sideroforos (p/a) | + | + |
| Chemotaxonomic features | | |
| iso-C_15:0_ | 30.96 % | ND |
| anteiso C_15:0_ | 8.25 % | ND |
| iso-C_16:0_ | 7.13 % | ND |
| C_16:0_ | 4.55 % | 31.53 % |
| iso-C_17:0_ | 7.61 % | ND |
| Sum In Feature 3 | 7.25 % | 25.65 % |
| Sum In Feature 8 | ND | 18.59 % |
| Genomic characterization | | |
| Genome size | 5,385,342 bp | 6,158,284 bp |
| %GC | 35.30 % | 61.10 %. |

***Table S3****. Physicochemical composition of ORGAON® PK before and after UV-C sterilisation (15 min, 265 nm)*

| **Parameter**  **(mean ± SD, n = 3)** | **OPK (crude)** | **OPK_ST (UV-C)** | **% change** |
| --- | --- | --- | --- |
| Total humic extract (%) | 6.79 ± 0.04 | 6.71 ± 0.06 | –1.2 % |
| Fulvic acids (%) | 6.79 ± 0.04 | 6.67 ± 0.05 | –1.8 % |
| Dissolved organic C (g L⁻¹) | 55.3 ± 0.8 | 54.6 ± 1.1 | –1.3 % |
| NH₄⁺-N (mg L⁻¹) | 112 ± 5 | 111 ± 4 | –0.9 % |
| PO₄³⁻-P (mg L⁻¹) | 64 ± 3 | 63 ± 2 | –1.6 % |

***Table S4****. Baseline physicochemical properties of the experimental soil (0–20 cm) at Villanueva de San Mancio (Valladolid, Spain) before treatment application*

| Parameter | Value (mean ± SD) | Method |
| --- | --- | --- |
| Texture | 37 % sand, 34 % silt, 29 % clay | Hydrometer |
| pH (H₂O, 1:2.5) | 8.1 ± 0.1 | ISO 10390 |
| Electrical conductivity | 0.24 ± 0.03 dS m⁻¹ | ISO 11265 |
| Organic C | 12.1 ± 0.5 g kg⁻¹ | Walkley–Black |
| Total N | 1.11 ± 0.07 g kg⁻¹ | Kjeldahl |
| C : N ratio | 10.9 ± 0.4 | – |
| CaCO₃ equivalent | 19.2 ± 1.3 % | Bernard calcimetry |
| Olsen P | 12 ± 2 mg kg⁻¹ | ISO 11263 |
| Exchangeable K | 195 ± 18 mg kg⁻¹ | NH₄OAc, AAS |

***Table S5.*** *Effects of fertiliser matrix and PGPB inoculation on rhizosphere functional-diversity indices (EcoPlate assay).*

| **p-value** | **Index** | **Test** |
| --- | --- | --- |
| 0.085 | Shannon_H | ANOVA (normal distribution) |
| 0.102 | Simpson_D | ANOVA (normal distribution) |
| 0.213 | Evenness_J | ANOVA (normal distribution) |
| 0.526 | Richness_S | Kruskal-Wallis (not normal distribution) |

***Table S6.*** *Pairwise comparison of α-diversity (Shannon index) between treatments.*

| **Facet** | **Group A** | **Group B** | **A** | **B** |  |  |  |
| --- | --- | --- | --- | --- | --- | --- | --- |
|  | **(see facet)** | **(see facet)** | **Median of** | **Median of** | **Test-statistic** | **p-value** | **q-value** |
| Bacteria | C1 | C2 | 9.02 | 9.03 | 22 | 0.32 | 0.70 |
| Bacteria | C1 | C0 | 9.02 | 9.06 | 28 | 0.72 | 0.81 |
| Bacteria | C2 | C0 | 9.03 | 9.06 | 35 | 0.79 | 0.81 |
| Fertiliser | CF | OPK | 9.20 | 9.15 | 20 | 0.81 | 0.81 |
| Fertiliser | CF | OPK_ST | 9.20 | 7.92 | 32 | **0.02** | **0.07** |
| Fertiliser | CF | W | 9.20 | 9.23 | 16 | 0.81 | 0.81 |
| Fertiliser | OPK | OPK_ST | 9.15 | 7.92 | 34 | **0.008** | **0.03** |
| Fertiliser | OPK | W | 9.15 | 9.23 | 12 | 0.39 | 0.70 |
| Fertiliser | OPK_ST | W | 7.92 | 9.23 | 0 | **0.002** | **0.01** |

Median values refer to Shannon H; statistics are Mann–Whitney U for bacterial treatments and Kruskal-Wallis pairwise contrasts for fertiliser matrices. A q-value < 0.10 (bold) denotes significance after FDR correction.

***Table S7.*** *Top discriminant KEGG orthologues (KOs) for each treatment and their predicted functions.*

| Tratamiento | KO | Función predicha (KEGG) |
| --- | --- | --- |
| WC1 | K12057 | conjugal transfer pilus assembly protein TraF |
|  | K16439 | reductase EvaE |
| WC2 | K01893 | asparaginyl-tRNA synthetase [EC:6.1.1.22] |
| OPKC0 | K08965 | 2,3-diketo-5-methylthiopentyl-1-phosphate enolase [EC:[5.3.2.5](https://www.genome.jp/entry/5.3.2.5)] |
| OPKC1 | K00455 | 3,4-dihydroxyphenylacetate 2,3-dioxygenase [EC:[1.13.11.15](https://www.genome.jp/entry/1.13.11.15)] |
| OPKSTC1 | K11960 | urea transport system permease protein |
|  | K02778 | glucose PTS system EIIB component [EC:[2.7.1.199](https://www.genome.jp/entry/2.7.1.199)] |
|  | K16922 | putative peptide zinc metalloprotease protein |
|  | K04772 | serine protease DegQ [EC:3.4.21.-] |
|  | K07070 | uncharacterized protein |
|  | K00797 | spermidine synthase [EC:[2.5.1.16](https://www.genome.jp/entry/2.5.1.16)] |
|  | K00822 | beta-alanina-piruvato transaminasa [EC: [2.6.1.18](https://www.genome.jp/entry/2.6.1.18) ] |
|  | K09971 | general L-amino acid transport system permease protein |
| OPKSTC2 | K06436 | spore coat assemly protein |
|  | K07148 | uncharacterized protein |


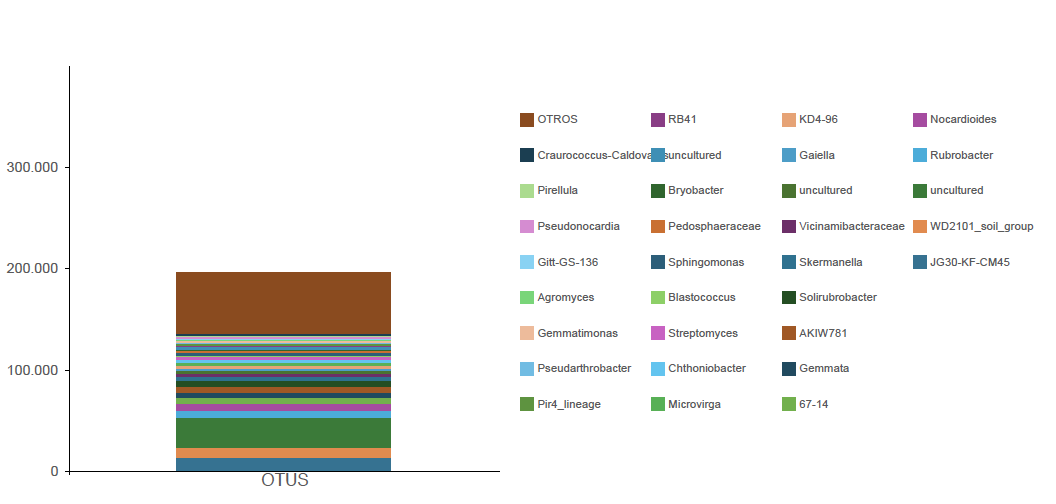


***Fig. S1. Taxonomic profile of the crude ORGAON® PK residue before inoculation.*** *Relative abundance of the 30 most represented bacterial genera (amplicon sequence variants, V3–V4 16S rRNA) detected in triplicate aliquots of the residue used as fertiliser carrier. Bars are stacked by genus (colour-coded in the legend); the category “OTROS” groups taxa contributing < 1 % of total reads each. Dominant lineages are typical soil saprotrophs—Rubrobacter, Nocardioides, Pirellula, Bryobacter and Sphingomonas—while human/animal pathogens were absent (< 0.01 % reads), supporting the biosafety of the starting matrix.*

| 1. **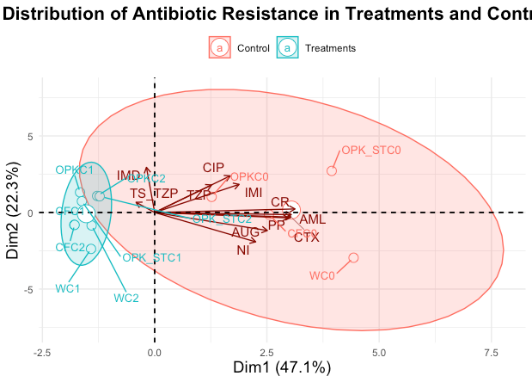***Variance table for the first eleven PCs*  \| **Variance** \| PC1 (AML) \| PC2 (AUG) \| PC3 (PP) \| PC4 (CR) \| PC5 (CTX) \| PC6 (TZP) \| PC7 (TS_TZP) \| PC8 (IMI) \| PC9 (IMD) \| CP10 (NI) \| PC11 (CIP) \| \| --- \| --- \| --- \| --- \| --- \| --- \| --- \| --- \| --- \| --- \| --- \| --- \| \| E.V% \| 47.1 \| 22.3 \| 10.5 \| 8.0 \| 5.0 \| 2.5 \| 1.8 \| 1.2 \| 0.8 \| 0.5 \| 0.3 \| \| A.V% \| 47.1 \| 69.4 \| 79.9 \| 87.9 \| 92.9 \| 95.4 \| 97.2 \| 98.4 \| 99.2 \| 99,7 \| 100 \|  1. **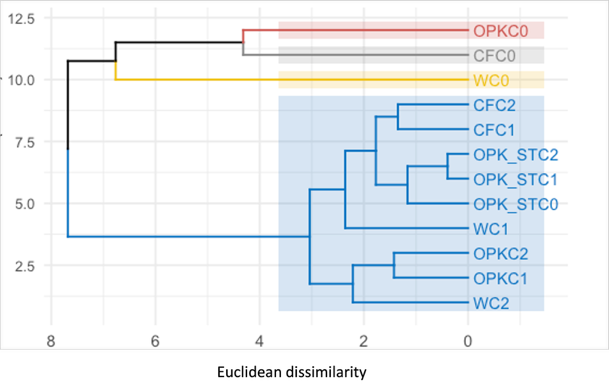** *PCA biplot of MICs c) Ward dendrogram based on z-scored MICs* 2. *One-way ANOVA F- and p-values for each antibiotic*  \| **ANOVA** \| AML \| AUG \| PP \| CR \| CTX \| TZP \| TS_TZP \| IMI \| IMD \| NI \| CIP \| \| --- \| --- \| --- \| --- \| --- \| --- \| --- \| --- \| --- \| --- \| --- \| --- \| \| F-Statistic \| 19.54 \| 19.04 \| 29.72 \| 1.06 \| 19.6 \| 1.71 \| 0.04 \| 3.35 \| 0.01 \| 3.63 \| 4.34 \| \| p-Value \| **0.001** \| **0.001** \| **0.0002** \| 1.74 \| **0.001** \| 0.21 \| 0.84 \| 0.09 \| 0.92 \| 0.08 \| 0.06 \|  1. *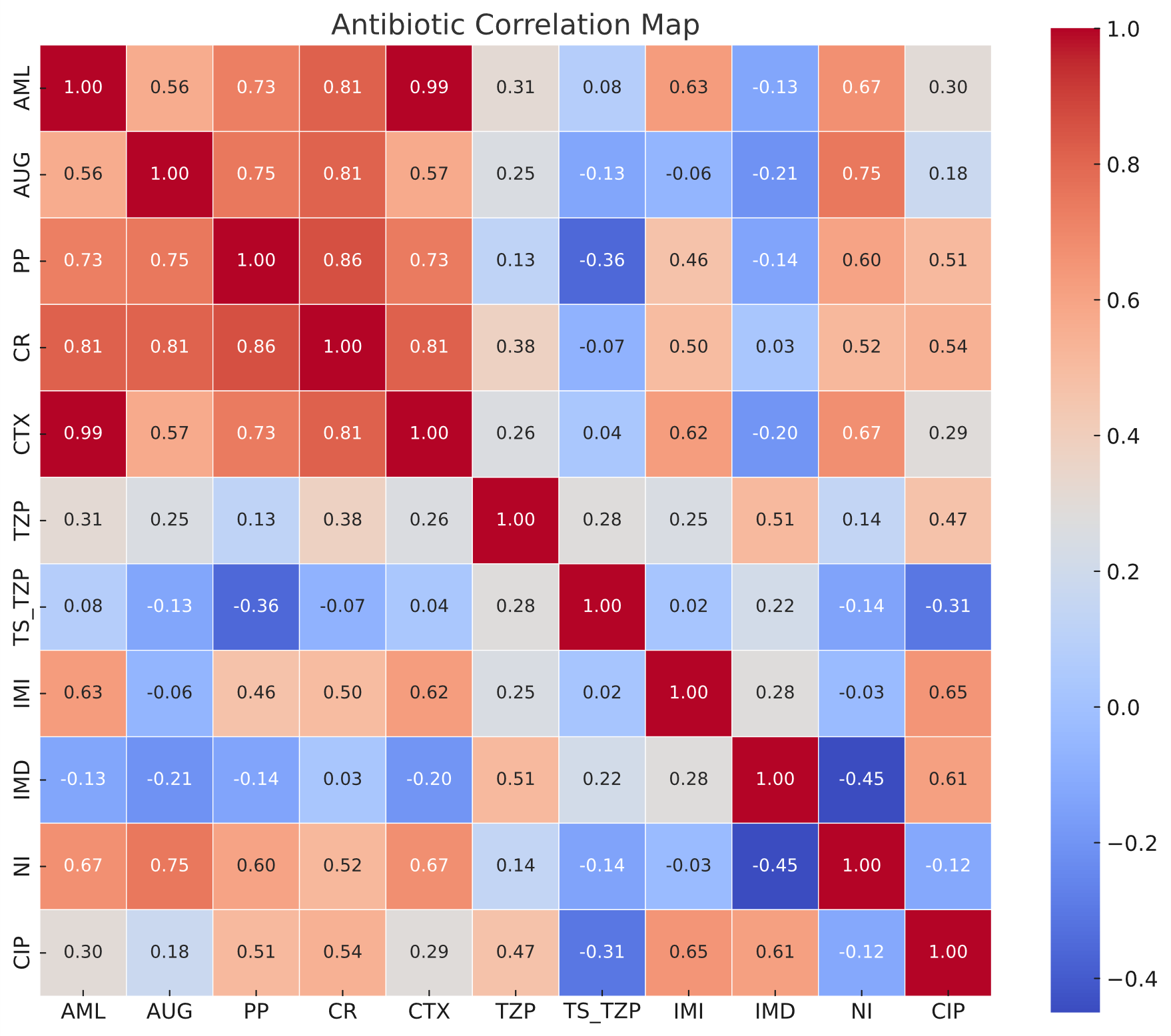 Pearson correlation matrix of MICs* |
| --- | --- | --- | --- | --- | --- | --- | --- | --- | --- | --- | --- | --- | --- | --- | --- | --- | --- | --- | --- | --- | --- | --- | --- | --- | --- | --- | --- | --- | --- | --- | --- | --- | --- | --- | --- | --- | --- | --- | --- | --- | --- | --- | --- | --- | --- | --- | --- | --- | --- | --- | --- | --- | --- | --- | --- | --- | --- | --- | --- | --- | --- | --- | --- | --- | --- | --- | --- | --- | --- | --- | --- | --- |

***Fig. S2****. Integrated analysis of community-level antibiotic resistance (cenoantibiogram). a) Variance table for the first eleven PCs; EV % = variance explained by each PC, AV % = cumulative variance. b) PCA biplot of MICs; symbols denote treatments (red = no inoculant, blue = PGPB), ellipses = 95 % confidence. Vectors correspond to the eleven tested antibiotics: AML amoxicillin, AUG amoxicillin + clavulanate, PP piperacillin, CR cefpirome, CTX cefotaxime, TZP piperacillin + tazobactam, TS_TZP trimethoprim–sulphonamide + tazobactam, IMI imipenem, IMD imipenem + EDTA, NI nitrofurantoin, CIP ciprofloxacin. c) Ward dendrogram based on z-scored MICs; red branches = strain-free controls, blue = inoculated treatments. d) One-way ANOVA F- and p-values for each antibiotic (α = 0.05). e) Pearson correlation matrix of MICs (red = positive, blue = negative). Boxes sharing the same shading indicate highly correlated β-lactam responses (r > 0.60).*

a)


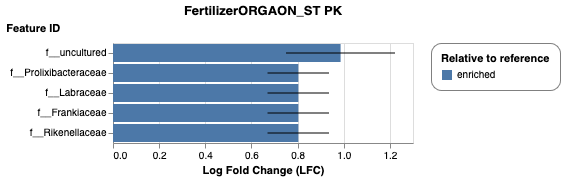


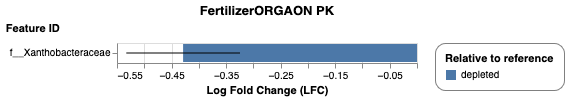


c)

b)


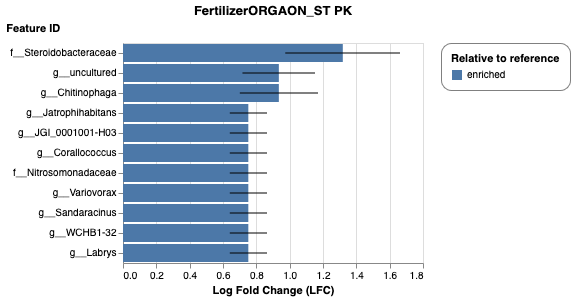


***Fig. S3. Differentially abundant bacterial taxa identified by ANCOM-BC in response to the organic-residue fertilisers.*** *(a) Family-level log₂ fold-changes (LFC) for the UV-sterilised residue (OPK_ST) versus the pooled water + mineral control (WC + CF). Five families Prolixibacteraceae, Labrysaceae, Frankiaceae, Rikenellaceae and an uncultured lineage—were significantly enriched (LFC ≈ +0.8 to +1.0, false-discovery rate [FDR] < 0.05). (b) Family-level comparison for the crude residue (OPK) revealed a single significant depletion of Xanthobacteraceae (LFC ≈ –0.4). (c) Genus-level profile for OPK_ST showed eleven enriched genera, headed by the polymer degraders Chitinophaga and an unclassified Steroidobacteraceae genus, together with the nitrifier proxies Variovorax and Nitrosomonadaceae; LFC values range from +0.6 to +1.5. No significant genera were detected for crude OPK, the mineral fertiliser or the water control. Bars represent bias-corrected LFC ± standard error; positive values denote enrichment, negative values depletion relative to the control baseline. Only taxa meeting the significance threshold (FDR-adjusted p < 0.05) are displayed, and the absence of a panel indicates that no significant features were detected at that taxonomic rank for the treatment concerned.*


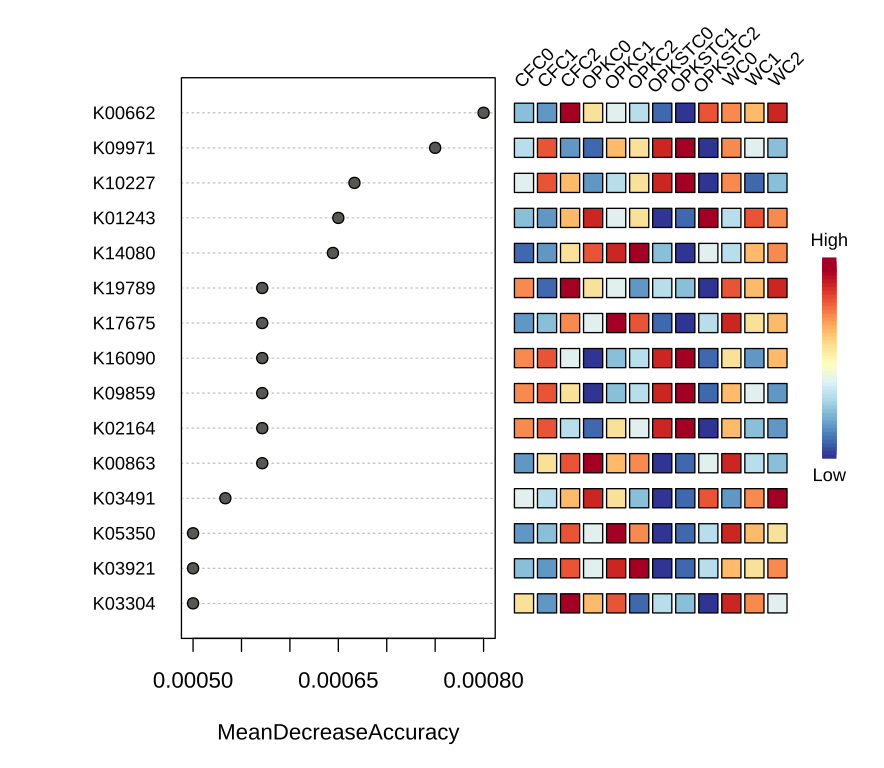


***Fig. S4. Discriminant KEGG orthologues identified by random-forest analysis.*** *Left: mean decrease in classification accuracy (MDA) for the 15 most informative KEGG orthologues (KOs) distinguishing the 12 fertiliser × inoculant treatments (10-fold cross-validation). Right: Z-score-normalised abundance of each KO across treatments; red = high, blue = low. The model was trained on PICRUSt2-predicted KO counts (centre-log-ratio transformed) and achieved 82 % overall accuracy.*
